# Turbulence-driven shifts in holobionts and planktonic microbial assemblages in St. Peter and St. Paul Archipelago, Mid-Atlantic Ridge, Brazil

**DOI:** 10.3389/fmicb.2015.01038

**Published:** 2015-10-02

**Authors:** Ana Paula B. Moreira, Pedro M. Meirelles, Eidy de O. Santos, Gilberto M. Amado-Filho, Ronaldo B. Francini-Filho, Cristiane C. Thompson, Fabiano L. Thompson

**Affiliations:** ^1^Laboratory of Microbiology, Institute of Biology, Federal University of Rio de JaneiroRio de Janeiro, Brazil; ^2^Fundação Centro Universitário Estadual da Zona Oeste (Uezo)Rio de Janeiro, Brazil; ^3^Diretoria de Pesquisa Científica, Instituto de Pesquisas Jardim Botânico do Rio de JaneiroRio de Janeiro, Brazil; ^4^Department of Environment and Engineering, Federal University of ParaíbaRio Tinto, Brazil

**Keywords:** metagenomics, Atlantic Ocean, oceanic islands, Scleractinia, *Madracis decactis*

## Abstract

The aim of this study was to investigate the planktonic and the holobiont *Madracis decactis* (Scleractinia) microbial diversity along a turbulence-driven upwelling event, in the world's most isolated tropical island, St Peter and St Paul Archipelago (SPSPA, Brazil). Twenty one metagenomes were obtained for seawater (*N* = 12), healthy and bleached holobionts (*N* = 9) before, during and after the episode of high seawater turbulence and upwelling. Microbial assemblages differed between low turbulence-low nutrient (LLR) and high-turbulence-high nutrient (HHR) regimes in seawater. During LLR there was a balance between autotrophy and heterotrophy in the bacterioplankton and the ratio cyanobacteria:heterotrophs ~1 (C:H). *Prochlorales*, unclassified Alphaproteobacteria and Euryarchaeota were the dominant bacteria and archaea, respectively. Basic metabolisms and cyanobacterial phages characterized the LLR. During HHR C:H < < 0.05 and Gammaproteobacteria approximated 50% of the most abundant organisms in seawater. *Alteromonadales, Oceanospirillales*, and Thaumarchaeota were the dominant bacteria and archaea. Prevailing metabolisms were related to membrane transport, virulence, disease, and defense. Phages targeting heterotrophs and virulence factor genes characterized HHR. Shifts were also observed in coral microbiomes, according to both annotation–indepent and -dependent methods. HHR bleached corals metagenomes were the most dissimilar and could be distinguished by their di- and tetranucleotides frequencies, Iron Acquision metabolism and virulence genes, such as *V. cholerae*-related virulence factors. The healthy coral holobiont was shown to be less sensitive to transient seawater-related perturbations than the diseased animals. A conceptual model for the turbulence-induced shifts is put forward.

## Introduction

Marine microbial communities are recognized as engines of globally important processes, such as the marine carbon, nitrogen and sulfur cycles (Falkowski et al., 2008; Fuhrman, 2009). Only recently with the introduction of molecular techniques have satisfactory descriptions of natural microbial assemblages been generated (Fierer and Jackson, 2006; Rusch et al., 2007; Costello et al., 2009). Nevertheless, most marine ecosystems are understudied. As a result, there is limited information on the diversity of microbial assemblages in changing environments and on the environmental drivers of microbial diversity shifts (Karl, 2002).

Nutrient dynamics in the sea is inextricably linked to variations in physical processes. Either enhanced nutrient delivery from turbulent mixing or upwelling, or enhanced stratification can lead to shifts in microbial assemblages, with significant consequences for nutrient cycling (Cullen et al., 2002). Episodic mixing events must occur in order to balance supply and demand (Hayward, 1987, 1991). Because open-ocean microbial assemblages are metabolically active with a potential for relatively high specific growth, they are poised to respond quickly and effectively to environmental perturbations (Karl, 2002). A model of turbulence-nutrients regimes decoupled characteristics and adaptations of phytoplankton assemblages and how they relate to food web structure: (i) high turbulence—low nutrients: low biomass, slow turnover, adaptations for efficient use of light and nutrients (e.g., iron-limited, high latitude waters); (ii) LLR: smaller cells, high turnover, competition for nutrients, retention by recycling (microbial loop); (iii) low turbulence—high nutrients: larger cells, higher biomass, slower turnover, selective pressure to sequester nutrients and minimize losses (e.g., noxious toxic blooms); and (iv) HHR: larger cells, higher biomass, transient, and self-limiting selection for rapid growth (e.g., diatoms). According to this model, the microbial loop is present in all regimes but it dominates the biomass in the low turbulence—high nutrients regime (Cullen et al., 2002).

Some physical mechanisms that vertically supply nutrients from below to the oligotrophic oceanic surface layers are: (i) internal waves and tides, (ii) cyclonic mesoscale eddies, (iii) wind-driven Ekman pumping, and (iv) atmospheric storms (Karl, 1999). Internal waves are ubiquitous in deep-ocean environments (Garrett and Munk, 1972), and it appears that the high vertical shear of low-frequency internal waves contributes to occasional pulses of vertical mixing (Gregg et al., 1986; Sherman and Pinkel, 1991). Stochastic events, that may be short-lived, are of major ecological significance. They are undoubtedly undersampled by ship-based observation programs (Platt et al., 1989). Even a monthly sampling schedule, as adopted in the Hawaii Ocean Time-series (HOT) program, is considered too infrequent to register important but intermittent nutrient injections (Karl, 1999). Furthermore, most studies are restricted to the Pacific, Caribbean, and North Atlantic.

St. Peter and St. Paul Archipelago (SPSPA) are the smallest and most isolated tropical islands in the world. It comprises the most seaward Brazilian oceanic islands (~1000 km from mainland) and the unique on the northern hemisphere, lying 100 km off the equator (00°55′N; 29°22′W). Data from the Sea-viewing Wide Field-of-view Sensor (SeaWiFS) evinces the chlorophyll pattern characteristic of mesotrophic waters (0.1–0.3 mg.m^−3^ Chla) (Supplementary Figures 1A,B), according to the classification of Shushkina et al. (1997). SPSPA is located in the biogeochemical Western Tropical Atlantic Province (WTRA) (Longhurst et al., 1995), under the influence of the Intertropical Convergence Zone. Its formed by minute summits of the Mid-Atlantic Ridge (MAR) within the St Paul Fault Zone (FZ), between the South American and the African Plates (Supplementary Figures 2A,B), where seismicity is frequent (Campos et al., 2010). The islets are devoid of shore and consist entirely of steep scarpments extending to 60–100 m depth, with the most limited area of shallow habitat among oceanic islands (~200 m) (Robertson, 2001). Within 2 km diameter bathyal depths are reached and within 5 km depths fall within the abyssal range (−3.600 to 5.000; Supplementary Figure 2B). The South Equatorial Current (SEC) flows E-W superficially and the Atlantic Equatorial Undercurrent (EUC) flows W-E at 60–100 m depth (Edwards and Lubbock, 1983). The latter is one of the fastest, varying and least predictable among the Atlantic currents, which reaches 120 cm.seg^−1^ above the thermocline (Philander, 1986). A permanent thermocline may prevent deep water masses to emerge (Macedo et al., 2009) nevertheless the eventual presence of deep water *Pleurommama* spp. and *Heterorhabdus* spp at the shallow layer during the diurnal period is contradictory to the former assumption (Macedo-Soares et al., 2009). There are no reports on local hydrodynamics but, seemingly, intermittent vertical flow of deep water masses should result from the violent interaction with the geomorphology—MAR—a perpendicular barrier to the currents. In principle, the friction between the SEC and EUC masses, which flow in opposing directions, should promote and intensify episodic extensions of the thermohaline to the photic zone.

Moreira et al. (2014) reported a significant enrichment process along an 8 days period during which an ever-growing turbulence with surge pulses was observed in SPSPA. The process occurred along the lunar phase from crescent to full moon. The work performed the first (and unique to date) characterization of the culturable heterotrophic bacterial community of SPSPA. Bacterial counts (colony forming units, CFU) correlated positively with nutrients in seawater, which in turn correlated positively with turbulence—energy and frequency of the surges. In the present work we analyzed the metagenomic composition and diversity of both the planktonic microbial assemblages and in the scleractinian coral *M. decactis* along the same period in the same locale. The aim was to characterize the microbial diversity during an upwelling-driven nutrient enrichment. We did not expect to find bleached corals in SPSPA. The coral holobionts were targeted in this survey to investigate whether there was a correlation between the metagenomic features and seawater parameters (vibrio counts, nutrients, bacterioplankton composition). Sampling was performed before, during and after a turbulence surge. It was a short-lived event, which is locally recurrent. We analyzed 21 metagenomic samples of seawater (*n* = 12), healthy and bleached corals (*n* = 9). The findings are summarized in a model of the physico-chemical-biological dynamics in SPSPA, where a cyclic recurrent pattern with extreme regimes of low turbulence-low nutrients (LLR) and high turbulence-high nutrients (HHR) is hypothesized to contribute to structure the marine ecosystem in that barren archipelago.

## Materials and methods

### Field sampling (performed by Moreira et al., 2014)

In brief, sampling was performed by SCUBA diving at the Sub-caulerpa zone (mesophotic), according to the zonation of (Edwards and Lubbock, 1983; Moreira et al., 2014). The survey took place along the NW side of the archipelago from the inlet (ca 4500 m^2^) to Belmonte islet's contiguous vertical wall. The satellite view and topograghy of the inlet are shown in Supplementary Figures 1C, 2B, respectively. The turbulence surge occurred along the lunar phase from crescent (14/Sep/2010) to full moon (22/Sep/2010). The peak of the surge overlapped with cloudiness, wind and rain. Rain and swash flushed guano, a possible additional source of nutrients (Gagnon et al., 2013), from the cays into the bay. Samples were obtained from the onset (LLR, *t*_1_ = 14/Sep/2010), while enhancing (*t*_2_ = 15; *t*_3_ = 18/Sep/2010, HHR) and almost to recovery of LLR condition, or recovery for short (*t*_4_ = 22/set/2010). During climax (20–21/Sep/2010) the strong vortex precluded diving (Supplementary Video 1). Henceforth samples will be referred as 14, 15, 18, and 22. In total, 12 colony fragments (10 × 10 cm) of *M. decactis* (healthy: *n* = 8; bleached: *n* = 4) were collected with hammer and chisel. On days 14 and 15 bleached corals were not found. Coral samples processed for metagenomics were: (i) healthy corals (Mad): 14 (*n* = 1), 15 (*n* = 2), 18 (*n* = 1), and 22 (*n* = 1); and (ii) bleached corals (MadBle): 18 (*n* = 2) and 22 (*n* = 2). Seawater was sampled from the water column immediately above the corals (<1 m) (4 samples: 14, 15, 18, and 22; 20 L/sample; 3 sterivex/sample). All samples were taken immediately to the Scientific Staion (SS) Laboratory, 20 m from the pier (Supplementary Figure 1D; view of the pier and the SS from the water at LLR). Seawater was filtered. Filters and coral samples were preserved in liquid nitrogen until DNA extraction (no longer than 3 months after).

### *Madracis decactis* (Lyman, 1859) (scleractinia: pocilloporideae)

Is a colonial zooxanthellate scleractinian coral. It has a variable bathymetric distribution, from 3 to 30 and up to 100 m (Neves and Johnsson, 2009). It is widespread in Brazil (N to SE), in Caribe, Gulf of Mexico and locally found in the Southeastern Atlantic (West Africa) (World Register of marine Species—WORMS: www.marinespecies.org). Free-living colonies of *M. decactis* display an unique formation off southern Brazil, at Galé Island. Spheroid shape, a.k.a. circumrotatory colonies, form the first corallith site discovered in the subtropical South Atlantic Ocean (SAO), at 6–15 m depth over 3400 m^2^ (coralreeefs-2012). In São Paulo (SE Brazil) it's a major contributor of reef structures, where bleaching has been seriously affecting its populations (Migotto, 1997), adding interest to the study of this coral species. In SPSPA, its one of the two scleractinian species locally found, mostly at the mesophotic zone. Healthy, bleached and with scars left by fish predation (*Stegastes sanctipauli*, Pomacentridae; *Halichoeres radiatus*, Labridae) in SPSPA are shown in Supplementary Figure 3.

### Seawater: temperature, nutrients, and microbial abundance

Environmental parameters were analyzed by standard oceanographic methods with at least three replicates for each parameter and determined by Moreira et al. (2014). Temperature was recorded *in situ* with a HOBO UA-002-64/date Logger and UEMIS dive computer from 5, 15, 33, 45, and 65 m depth (*n* = 5 for each depth), during September/2010 and June/2011 (published in Crespo et al., 2014). Environmental data from (Moreira et al., 2014) and temperature from (Crespo et al., 2014) are summarized in Supplementary Figure 4, for aid in data interpretation.

### Metagenomic DNA extraction

Corals' DNA extraction was performed as in Trindade-Silva et al. (2012). Seawater was sequentially pre-filtered (100 and 20 μm) by gravity and then filtered on the Sterivex (0.22 μm) by positive pressure using Niskin system (2 L/Sterivex). The microbes collected at Sterivex filters were preserved with SET buffer (20% sucrose, 50 mM EDTA and 0.5 mM Tris–HCl). Metagenomic DNA extraction was performed using lysozyme (1 mg/mL) for 1 h at 37°C. Then, proteinase K (0.2 mg/mL) and sodium dodecyl sulfate (SDS) (1% v/v) were added and incubated (55°C; 60 min) under agitation. The lysate was rinsed into SET buffer (1 mL). Organic extraction was performed with one volume of phenol:chloroform:isoamyl alcohol (25:24:1). DNA precipitation was performed with ethanol (2,5 volumes) and amnonium acetate (0.7 M) at −20°C overnight. After centrifugation the pellet was washed twice with ethanol (70%) and air-dried. Elution was done in TE buffer (1X). Three libraries were prepared for each Sterivex and coral sample and pyrosequenced subsequently.

### Metagenomic library construction

Metagenomes were obtained by pyrosequencing technology using a 454 GS Junior instrument (Roche) (Margulies et al., 2005). Shotgun libraries were generated with 500 ng of whole metagenome samples, sheared into fragments by nebulization. End-repair and adaptor ligation were performed using GS FLX Titanium kit (Roche). Quality control and quantification were performed with Agilent 2100 Bioanalyzer (Agilent Technologies) and TBS 380 Fluorometer (Turner Biosystems), respectively. After the libraries construction, approximately 10^6^ molecules/metagenome were denatured and amplified by emulsion PCR.

### Metagenomic data analysis

Raw sequences were submitted to quality control using PRINSEQ Standalone Lite (version 0.20.4; available at http://sourceforge.net/projects/prinseq/files/). We analyzed 21 metagenomic samples of seawater (*n* = 12) and corals (*n* = 9) from 4 days (*t*_1_ = 14, *t*_2_ = 15, *t*_3_ = 18, *t*_4_ = 22, of set/2010) along the enrichment process (*t*_4_−*t*_1_ = 8 days). Annotation was performed by Meta-Genome Rapid Annotation using Subsystems Technology (MG-RAST) server (Meyer et al., 2008) version 3.0, using (SEED) Subsystems Technology and the GenBank database for functional and organismal classifications, respectively. For this purpose, all BLAST queries were conducted with a maximum cutoff *E-*value 0.00001, a minimum identity of 60%, and a minimum alignment length of 20 measured in aa for protein and bp for RNA databases.

### Metagenomes comparison trough annotation-independent analysis

Dinucleotide odds ratio and Karlin distances (δ) between the metagenomes, based on the dinucleotide relative abundances differences (according to Karlin et al., 1997), were calculated using Perl scripts as in Willner et al. (2009). The values in the Karlin matrix were multiplied by a 1000 for easier comparison. Tetranucleotide frequencies were calculated using a Perl script as in Albertsen et al. (2013). The divergence between the observed and expected tetranucleotide frequencies was transferred into *z*-scores and pairwise comparison of the metagenomic sequences was performed by computing the Pearson's correlation coefficients of the *z*-scores, both through Python scripts, according to (Teeling et al., 2004). Seawater metagenomes (Sw-14, -15, -18, and -22) were compared among each other, as well as coral metagenomes (Mad14, Mad15, Mad18, MadBle18, Mad22, MadBle22).

### Phage detection

The 21 metagenomic libraries were searched for phages using the PHAge Search Tool (PHAST), available at http://phast.wishartlab.com (Zhou et al., 2011). Briefly, pyfasta 0.5.2 (available at http://pypi.python.org/pypi/pyfasta/) was used to split metagenomes into smaller subsets without splitting individual fasta entries, after which PHAST was used to phage search. The tool provides an ensemble of ORF prediction and translation (via GLIMMER; Salzberg et al., 1998), protein identification (via BLASTP; Altschul et al., 1997), phage sequence identification (via BLAST matching to a specific database), tRNA identification (using tRNAscan-SE; Lowe and Eddy, 1997), attachment site recognition (with ARAGORN; Laslett and Canback, 2004) and gene clustering density measurements using density-based spatial clustering of applications with noise (DBSCAN; Ester et al., 1996), and sequence annotation text mining. PHAST's database encloses protein sequences from two sources: the NCBI phage database and the prophage database (Srividhya et al., 2006). Specific keywords (e.g., “protease,” “integrase,” and “tail fiber”) are used for screening. Matched phage or phage-like sequences with *E* < 0.0001 are saved as hits and their positions tracked for subsequent evaluation for local phage density by DBSCAN. Phage schemes shown in Supplementary Figures 11–18 were generated with PHAST.

### Sequence comparison to the virulence factor database

The 21 metagenomic libraries were compared to the virulence factor database (VFDB) (Chen et al., 2005) (http://www.mgc.ac.cn/VFs/) using BLASTX (*E* < 0.0001).

### Statistics

Statistical analysis was conducted using R Version 3.1.3 (Team, 2012) with a suite of packages. The comparison of the correlation coefficients of the *z*-scores obtained for the tetranucleotide frequencies was visualized through heatmaps using rpy2 and gplots (Gautier, 2008). An exploratory analysis aiming to correlate samples with nutrients' concentrations and metabolisms, according to the level 1 SEED classification, was performed by means of a principal component analysis (PCA) using the rda function of Vegan package (Oksanen et al., 2013). Abundance plots were drawn using the ggplot2 and reshape packages (Wickham, 2007; Wickham and Chang, 2009). The cluster analysis was performed with the APE package (Paradis et al., 2004) using Pearson correlation and ward distance.

### Sequence data

The metagenomic data that we generated are available in the MG-RAST v3 server (http://metagenomics.anl.gov/metagenomics.cgi) under the unique identifiers: 4461594.3, 4463932.3, 4463930.3, 4463939.3, 4463927.3, 4461593.3, 4463933.3, 4463931.3, 4463928.3, 4463926.3, 4463925.3, 4468639.3, 4486661.3, 4486665.3, 4486669.3, 4486662.3, 4486667.3, 4486668.3, 4486664.3, 4486663.3, and 4486666.3; and in the Brazilian Marine Biodiversity database (BaMBa) (pmeirelles.18.1).

### Sampling permit

Ministério do Meio Ambiente (MMA), Instituto Chico Mendes de Conservação da Biodiversidade (ICMBio) Number 10112-2.

## Results

In this study 21 metagenomic libraries were obtained from seawater (*n* = 12) and corals (*n* = 9) with a total of 446,129 sequences (2.13 × 10^8^ bp) (Table 1). Through annotation-independent analysis, samples Sw14, -15, and -22 were distinguished from -18 (Table 1). Average Karlin distances for pairs of seawater samples Sw14, -15, -18 and -22 defined three categories based on δ ranges. (i) δ < 11 grouped pairs 14-14 and 22-22, indicating high degree of genetic similarity; (ii) 11 < δ < 30 grouped pairs 15–15, 18–18, 14–15, 14–22, and 15–22, indicating intermediate genetic similarity, and (iii) δ> 30 grouped pairs 14–18, 15–18, and 18–22, indicating low genetic similarity. The most dissimilar samples (δ = 50.6) were Sw14 (LLR) and Sw18 (HHR) (Supplementary Figure 5). Similarly, average Karlin distances for coral samples Mad14, -15, -18, and -22 defined three categories. (i) δ < 11 grouped pairs 14–22, 14–15, 15–22, and 22–22; (ii) 11 < δ < 20 grouped pairs 15–15; and (iii) δ> 20 grouped pairs 14–18, 15–18, 18–22, and 18–18. Samples Mad18 were the most dissimilar in comparison with -14, -15, -22 and also among each other (Supplementary Figure 6). The same pattern was revealed by the analysis that resulted from the tetranucleotide frequencies estimated for seawater and coral metgenomes. Samples Sw18 (HHR) were the most dissimilar amongst seawater metagenomes (Supplementary Figure 7A) and metagenomes from HHR bleached corals (MadBle18) were the most dissimilar amongst the holobionts' metagenomes (Supplementary Figure 7B).

**Table 1:** **General features of the metagenomes**.

**MGRast Id**	**Sample**	Day-^*^ replicate	**Depht (m)**	**# reads**	**GC content avg. ± SE (%)**	**GC content sample avg. ± SE (%)**	**Read lenght ± SE (bp)**	**ORFs**	**ORFs Avg lenght ± SE (bp)**	**Taxonomic annotated ORFs**
**SEAWATER**										
4461594.3	Sw	14-1	35	20,442	40 ± 11	39.3 ± 0.6	361 ± 128	19,088	345.6 ± 142.0	9385
4463932.3	Sw	14-2	35	14,001	39 ± 11		364 ± 128	13,087	348.3 ± 143.1	6423
4463933.3	Sw	14-3	35	8621	39 ± 11		418 ± 120	8259	387.1 ± 149.9	4505
4463930.3	Sw	15-1	35	7606	41 ± 11	40.3 ± 0.6	379 ± 117	7297	357.4 ± 138.4	4283
4463929.3	Sw	15-2	35	15,746	40 ± 11		379 ± 119	15,044	358.6 ± 139.0	42,928
4463931.3	Sw	15-4	35	13,654	40 ± 10		434 ± 109	13,346	399.0 ± 148.0	9295
4463927.3	Sw	18-1	40	12,022	46 ± 11	46.3 ± 0.6	392 ± 108	11,698	368.0 ± 165.0	10,863
4468639.3	Sw	18-2	40	47,952	47 ± 10		427 ± 122	46,879	391.0 ± 161.7	34,973
4463928.3	Sw	18-3	40	19,451	46 ± 11		447 ± 86	19,228	412.1 ± 134.3	13,573
4461593.3	Sw	22-1	40	28,449	41 ± 11	40.7 ± 0.6	389 ± 113	27,445	365.4 ± 138.0	16,966
4463926.3	Sw	22-2	40	17,721	40 ± 10		429 ± 111	17,148	393.8 ± 149.0	11,029
4463925.3	Sw	22-3	40	19,312	41 ± 11		439 ± 100	18,786	403.7 ± 142.1	12,365
**CORALS**										
4486661.3	Mad	14	35	20,986	40 ± 07	–	434 ± 123	17,974	402.9 ± 156.8	1542
4486662.3	Mad	15-1	35	34,650	39 ± 05	40 ± 1.4	419 ± 139	30,109	394.9 ± 162.1	2030
4486663.3	Mad	15-2	35	29,172	41 ± 08		403 ± 109	23,895	379.2 ± 135.7	2869
4486664.3	Mad	18	40	24,249	39 ± 06	–	469 ± 100	21,019	432.5 ± 147.3	1413
4486665.3	MadBle	18-1	40	15,342	45 ± 10	46.5 ± 2.1	449 ± 98	11,527	414.4 ± 143.2	2799
4486666.3	MadBle	18-2	40	30,622	48 ± 11		369 ± 109	21,488	346.7 ± 132.2	7076
4486669.3	Mad	22	40	31,218	40 ± 08	–	428 ± 129	26,932	399.2 ± 158.5	2548
4486667.3	MadBle	22-1	40	38,869	39 ± 06	40 ± 1.4	463 ± 105	33,843	427.0 ± 149.9	2204
4486668.3	MadBle	22-2	40	16,044	41 ± 08		456 ± 105	13,662	420.6 ± 150.3	1313

* *Day of set/2010; Sw, seawater; Mad M. decactis; MadBle bleached M. decactis, Avg, Average; SE, standard error*.

**Figure 1:**
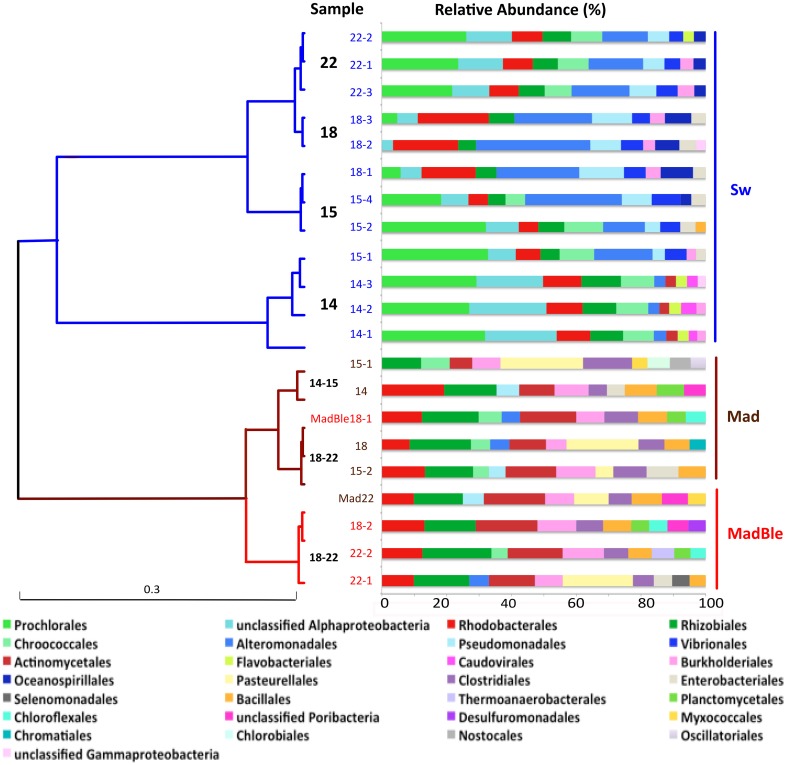
**Hierarchical clustering of the metagenomes using Order level of the taxonomical identification by MG-Rast**. The Pearson correlation and ward distance were used to create the dendrogram. The metagenomes from seawater, healthy and bleached *M. decactis* are indicated by the colors blue, brown, and red, respectively; and by the sampling day (of September/2010). Exceptions to the clustering of corals according to health status are detached (MadBle18-1 from bleached coral clustered within the healthy branch and Mad22 from healthy coral clustered within the bleached branch). The relative abundance of the 10 most abundant orders is presented at right.

To analyze the overall relationships among the most abundant taxa we performed a clustering analysis (Figure 1). The hierarchical clustering of the 21 metagenomes corroborated the binning based in sequence composition (di- and tetranucleotides frequencies). Two major branches split seawater and coral metagenomes. Two seawater branches were defined, with samples 14 (and Sw15-1) representing the LLR. Samples 15 (Sw15-2 and 4), 18 and 22 reflected the turbulence surge (Figure 1). Coral metagenomes were split into healthy and bleached (with only two exceptions: MadBle18-1 grouped into the healthy corals branch and Mad22 into the bleached corals branch). Healthy corals also showed a trend to group according to the enrichment gradient. The healthy corals branch split Mad14 and Mad15-1 from the remainders Mad15-2, Mad18, and Mad22.

### Taxonomic assignment of seawater metagenomes

Bacteria was the most abundant domain in all samples with overall average relative abundance of 97.0 ± 1.8% (Mean ± SD), followed by 1.11 ± 0.69% Eukarya (Supplementary Figure 8). Archaeal sequences accounted for ~0.4% of the overall sequences. The main groups were Euryarchaota (~58%) and Thaumarchaeota (21%). Viruses represented ~0.4% of sequences. The most abundant order was *Caudovirales* (~52%). The overall relative abundance of the most prevalent bacterial phyla were ~61% Proteobacteria, 32% Cyanobacteria followed by 2.4% Bacteroidetes (100% *Flavobacteriales*), 1.6% Firmicutes, and 1.3% Actinobacteria (Figure 1).

### Shifts in planktonic assemblages

**Figure 2:**
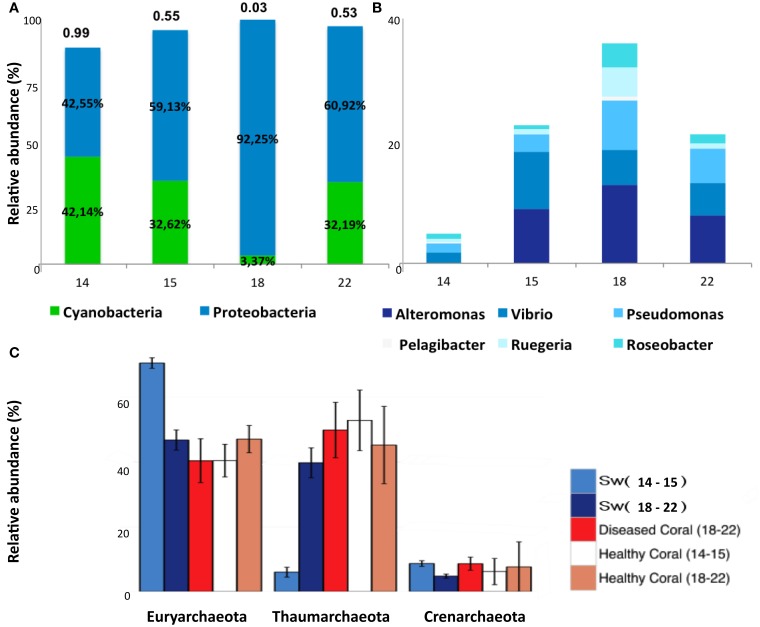
**Relative abundances of Cyanobacteria, Proteobacteria, and Archaea**. **(A)** Cyanobacteria and Proteobacteria relative abundances in samples Sw14, -15, -18, and -22. The ratio C:P is shown above bars. **(B)** Relative abundances of GammaProteobacteria (*Alteromonas, Vibrio, Pseudomonas*) and AlphaProteobacteria (*Candidatus pelagibacter, Ruegeria*, and *Roseobacter*) in samples Sw14, -15, -18, and -22. **(C)** Relative abundances of Euryarchaeota, Thaumarchaeota and Crenarchaeota in pooled samples seawater Sw14–15, Sw18–22; and *M. decactis* healthy indicated as Healthy coral (14–15) and (18–22); and diseased indicated as Diseased Coral (18–22).

The taxonomic classification of seawater samples revealed a clear difference between LLR and the remainder groups (Sw15, -18, -22). In all samples the dominant groups were Cyanobacteria (C) and Proteobacteria (P). At LLR (Sw14), these groups were equally abundant in seawater (C:P ~1). Along the turbulence gradient, Proteobacteria members increased and Cyanobacteria decreased to a vanishingly small proportion (Sw18, HHR; C:P < < 0.05), with a subsequent recovery (Sw22; C:P > 0.5). The highest proportion of Proteobacteria occurred at HHR (92%) (Figure 2A). At LLR Alphaproteobacteria presented the highest relative abundance (Alpha 28.3% and Gamma 25.1%; ANOVA, *P* < 0.05), whereas Gammaproteobacteria was dominant in all the following samples (Sw15, -18, and -22: Gamma 29.4% and Alpha 24.8%; *P* < 0.05) (Supplementary Figure 9). Proteobacterial groups whose relative abundances enhanced during enrichment were *Alteromonas, Vibrio* and *Pseudomonas* (Gamma)*; Ruegeria, Roseobacter*, and Candidatus *Pelagibacter* (Alpha) (Figure 2B). Cyanobacteria and unclassified Alphaproteobacteria (mostly SAR11) decreased correspondingly (Figure 1). *Oceanospirillales* was absent in the top ten rank at LLR, but appeared in Sw15-4 (2.5%), in HHR (5.6–7.3%), and Sw22 (2.5–2.7%). Archaeal groups also shifted dominance. The most abundant phyla in Sw14-15 was Euryarchaeeota, whereas in Sw18-22 it was Thaumarchaeota (Figure 2C).

### Functional assignment of seawater metagenomes

The overall most abundant subsystems were Protein Metabolism (9.8 ± 1.0%), Amino Acids and Derivatives (9.4 ± 0.7%), Carbohydrates (9.4 ± 0.7%), Cofactors, Vitamins, Prosthetic Groups, Pigments (7.2 ± 0.7%), and RNA Metabolism (5.2 ± 0.7%) (Supplementary Figure 10). The most abundant gene per metagenome was the TonB-dependent receptor, of the subsystem Iron Acquisition and Metabolism, in samples Sw18 (HHR) (Supplementary Table 1). A phage protein encoding gene was the most abundant gene in five samples: Sw22-1,2 (recovery) and Sw14-1,2,3 (LLR). Phage detection through specific databases (using PHAST) revealed that the most probable hosts were cyanobacteria for Sw14 and -22 (LLR and recovery, respectively), and Proteobacteria for the remainders (Sw15 and HHR), with only two exceptions (Table 2; Supplementary Figures 11–18). The range of unknown proteins was 17.9–42.4% (Sw18-2 and Sw14-1, respectively).

**Table 2:** **Abundance and characteristics of prophages found in the bacterioplankton in SPSPA**.

**Sample**	**Prophages #**	**GC content sample/phage (%)**	**Completeness**	**Length (Kb)**	**CDS #**	**Annotated phage proteins**	**Possible phage**
Sw14-1	1	40/42.5	incomplete	7.2	17	7	PHAGE_Synech_S_CRM01_NC_015569
Sw14-2	1	39/37.2	incomplete	6.2	16	9	PHAGE_Prochl_P_SSM3_NC_021559
Sw14-3	1	39/38.4	incomplete	8.5	22	5	PHAGE_Aeromo_Aes012_NC_020879
Sw15-1	1	41/42.5	incomplete	8.2	23	7	PHAGE_Bacill_G_NC_023719
Sw15-2	1	40/45.4	incomplete	10.9	30	10	PHAGE_Ectoca_siliculosus_virus_1_NC_002687
Sw18-2	1	47/46.3	incomplete	9.2	25	7	PHAGE_Bacill_G_NC_023719
Sw22-2	1	40/40.6	incomplete	8.8	24	6	PHAGE_Prochl_P_SSM3_NC_021559
Sw22-3	1	41/46.2	incomplete	8.1	21	5	PHAGE_Synech_S_SM2_NC_015279

*Sw, seawater; Mad M. decactis; MadBle bleached M. Decactis; #, number*.

### Shifts in metabolisms in seawater metagenomes

**Figure 3:**
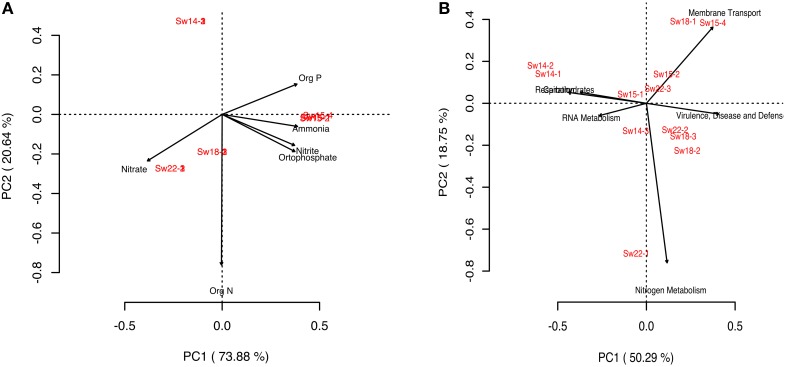
**Principal component analysis (PCA) diagrams**. **(A)** Diagram generated for the bacterioplankton samples and nutrients in seawater. **(B)** Diagram generated for the bacterioplankton samples and metabolisms in seawater.

To investigate the metabolic profile of planktonic dwelling microbes and to correlate differences with the enrichment gradient, we performed a PCA analysis (Figures 3A,B). Clustering of samples Sw14 (LLR) was not explained by nutrients concentrations, contrarily to the remainder samples (Figure 3A). Basic cell functions such as Carbohydrates and Respiration explained the clustering of samples Sw14 (LLR), whether enrichment samples Sw15 and Sw18 (HHR) clustered in response to Virulence, Disease, and Defence; Membrane Transport and Nitrogen Metabolism subsystems (Figure 3B). Samples Sw15 were grouped by the concentration of the nutrients organic phosphorous, ammonia, orthophosphate, and nitrite. The former three showed the highest concentration at Sw15 (Figure 3A, Supplementary Figure 4). HHR samples (Sw18) were coupled by the heterotrophic-characteristic metabolisms, Membrane Transport and Virulence, Disease and Defense. Accordingly, their most abundant gene was the TonB-dependent receptor (Virulence, Disease and Defense). Recovery samples (Sw22) were coupled by the concentration of nitrate (the highest, Figure 3A, Supplementary Figure 4) and Nitrogen Metabolism (Sw22-1; Figure 3B), correspondingly.

### Taxonomic assignment in *madracis decactis* metagenomes

Bacteria was the most abundant identified Domain in all samples with overall relative abundance of 57.15%, followed by Eukarya (39.63%). Archaeal sequences represented 1.92% and viruses 0.1%. Within the domain Eukarya, 33.59% of the sequences were Cnidaria (Supplementary Figure 19A). Main overall archaeal phyla were Euryarchaeota (45.61%), Thaumarchaeota (42.11%), and Crenarchaeota (10.96%). (Figure 2C). There were marked differences in comparison with seawater metagenomes (Figure 1). The most abundant bacterial phyla were Proteobacteria (~48% of the counts), Firmicutes (17%), Actinobacteria (10%), Bacteroidetes (6%), and Cyanobacteria (4%). The most abundant proteobacterial orders were *Rhizobiales* (16.29%), *Rhodobacterales* (12.44%); *Burkholderiales* (10.77%), *Pseudomonadales* (4.85%), *Pasteurellales* (4.82%), *Alteromonadales* (4.68%), *Desulfuromonadales* (4.38%), *Enterobacteriales* (4.12%), *Myxococcales* (4.01%), and *Chromatiales* (3.46%). Cyanobacteria was mostly represented by *Chroococcales* (50.23%), *Oscillatoriales* (24.35%), *Nostocales* (20.03%), and *Prochlorales* (5.24%). Interestingly, *Rhodobacterales, Rhizobiales, Actinomycetales, Burkholderiales*, and *Clostridiales* were abundant in healthy and diseased *M. decactis*.

### Shifts in microbial assemblages in *madracis decactis* metagenomes

At HHR bleached corals (MadBle18) showed more sequences affiliated to Proteobacteria than the healthy corals (Mad18) (Supplementary Figure 19B). The presence of *Pasteurellales* among the ten most abundant orders in five out of nine metagenomes (in Mad15, -18, and, -22 with 11–14% of counts) appeared to be a diagnostic feature (Figure 1). *Pasteurellales* was found in low relative abundance (<1%) in the remainder corals and in seawater metagenomes. Thaumarchaeota affiliates in all *M. decatis* samples (Mad14, -15, -18, and -22) approximated Sw18-22 abundance levels (Figure 2C). Cnidaria and Nematoda metagenomic sequences were more abundant in healthy and bleached corals, respectively (Supplementary Figure 8).

### Functional assignment in *madracis decactis* metagenomes

The overall most abundant subsystems were Carbohydrates (16.6 ± 2.8%), Amino Acids and Derivatives (14.3 ± 1.2%), Protein Metabolism (9.66 ± 1.6%), Cofactors, Vitamins, Prosthetic groups, Pigments (8.9 ± 1.6%), and DNA Metabolism (6.6 ± 2.4%) (Supplementary Figure 20).

### Shifts in metabolisms is *madracis decactis* metagenomes

To investigate major differences in metabolisms the corals' metagenomes were pooled (according to Figure 1 and Supplementary Figure 7B) in samples (i) healthy 14–15 (Mad14 and Mad15-1,2), (ii) healthy 18–22 (Mad18 and Mad22), and (iii) diseased 18–22 (MadBle18-1,2 and MadBle22-1,2). Seawater samples were included as reference (Sw14–15, Sw18–22). Prevalence of motility and chemotaxis in seawater compared to corals was the main difference observed between type samples. DNA metabolism prevailed in corals Mad18–22 (healthy and bleached) relative to the remainder samples (Supplementary Figure 20). DNA metabolism was the fourth most represented in corals. The overall most abundant gene (EC 2.1.1.72) in corals was affiliated to this subsystem, which was the most abundant gene in three metagenomes of the Mad18–22 group, and in none of the Mad14–15 group (Supplementary Table 1). The Iron Acquisition subsystem was investigated further by pooling coral samples according to health status in (i) healthy and (ii) diseased. The relative abundance range was lower in healthy corals (5.56–9.09%) than in diseased corals (5.15–16.36%) (*P* < 0.05). Iron Acquisition in *Vibrio* was the most abundant function in overall samples, with a relative abundance range of 18.2–83.3% (MadBle22-1 and Mad22, respectively). Healthy corals presented higher relative abundances (60–83%) than diseased corals (18.2–34.4%) for this function. Six functions were represented only in diseased corals: *Campylobacter* Iron Metabolism (6.3–40.0%); Heme, Hemin Uptake and Utilization Systems in GramPositives (0–20.0%); Siderophore Pyoverdine (0–18.2%), Transport of Iron (0–14.9%); Iron Acquisition in Streptococcus (0–10.3%) and Iron(III) Dicitrate Transport System (0–3.1%). Heme, Hemin Uptake and Utilization Systems in Gram-Negatives was overrepresented in diseased (16.2%) compared to healthy corals (7.3%) (*P* < 0.05).

### Shifts in the profiles of seawater- and *madracis decactis*- dwelling communities

The shift from autotroph:heterotroph-balance to offset was further investigated using virulence factors (VFs) as indicators of heterotrophy and risk or threat for corals. In total there were 21,230 significant similarities against the VFDB (Table 3). When normalized to library size, virulence genes were overrepresented in samples Sw 15, -18 (16.0–24.3) and, to a lesser extent in Sw22 (16.6–16.8) (recovery), compared to LLR (Sw14; 12.7–14.2); and in bleached (MadBle; 0.8–8.5) when compared to healthy corals (Mad; 0.6–3.1). We further investigated the iron uptake system, which is a nonspecific virulence system related to competition skills, and thus suitable to reflect the overall heterotrophic community. Iron related virulence genes comprised ~9% (*n* = 1817) of the total hits to the VFDB. Sw18 samples (HHR) presented the lower percentage of iron related genes relative to the total virulence hits per metagenome (*P* < 0.05), suggesting that other than iron uptake genes were most representative of the surplus heterotrophs. The VFDB lacks genes related to iron from vibrios (which was overly represented in healthy corals), but encompasses genes related to iron from *Haemophilus* (*Pasteurellales*). Among all virulence genes related to iron, ~33% (*n* = 606) fell into this category. *V. cholerae* related virulence genes, which confer infective skills, comprised ~6% (*n* = 1298) of the total virulence hits. The percentage range of these genes relative to total VFs per metagenome in seawater samples Sw14, -15, and -22 was 4.4–6.5% and in Sw18 (HHR) was 6.6–7.7%. Similarly, in healthy corals this range was 0.1–6.9%, and higher in diseased corals: 4.7–8.1% (Table 3). Heterotrophic populations that overgrew in response to turbulence-nutrient pulses were better represented by pathogenic (e.g., *V. cholerae*-related) than by non-specific (e.g., iron acquisition) VFs.

**Table 3:** **Abundances of virulence factor genes from microbes in the bacterioplankton and *M. decactis* in SPSPA**.

**Sample**	**Hits to Virulence Database #**	**Normalized to total bp (x 10^5^)**	**Iron-related virulence genes # (% of total virulence hits)**	***Haemophilus* (*Pasteurellales*) Iron-related virulence genes # (% of total iron related virulence genes)**	***Vibrio* (*V. cholerae*) related virulence genes # (% of total hits)**
**SEAWATER**					
Sw14-1	971	12.7	126 (13.0)	47 (37.3)	51 (5.2)
Sw14-2	698	13.1	81 (11.6)	45 (55.6)	35 (5.0)
Sw14-3	579	14.2	65 (11.2)	26 (40.0)	28 (4.8)
Sw15-1	547	18.2	62 (11.3)	19 (30.6)	24 (4.4)
Sw15-2	994	16.0	86 (8.7)	22 (25.6)	44 (4.4)
Sw15-4	1292	19.4	154 (11.9)	24 (15.6)	69 (5.3)
Sw18-1	1045	21.0	71 (6.8)	25 (35.2)	69 (6.6)
Sw18-2	4815	22.7	317 (6.6)	108 (34.0)	373 (7.7)
Sw18-3	2362	24.3	217 (9.2)	64 (29.5)	143 (6.1)
Sw22-1	1941	16.6	181 (9.3)	74 (40.1)	127 (6.5)
Sw22-2	1415	16.8	108 (7.6)	43 (39.8)	91 (6.4)
Sw22-3	1565	16.6	129 (8.2)	32 (24.8)	83 (5.3)
***M. DECACTIS***					
Mad14	145	1.5	11 (7.6)	10 (90.9)	1 (0.7)
Mad15-1	92	0.6	7 (7.6)	6 (86.0)	1 (1.1)
Mad15-2	435	3.1	28 (4.7)	9 (32.1)	30 (6.9)
Mad18	138	1.0	6 (4.3)	0 (0)	3 (2.2)
MadBle18-1	464	6.2	31 (6.7)	5 (16.1)	26 (5.6)
MadBle18-2	1020	8.5	87 (8.5)	29 (33.3)	55 (5.4)
Mad22	344	2.4	24 (7.0)	11 (45.8)	21 (6.1)
MadBle22-1	171	0.8	5 (2.9)	5 (100.0)	8 (4.7)
MadBle22-2	197	2.3	21 (10.7)	2 (9.5)	16 (8.1)
Total	21,230	238.1	1817 (8.6)	606 (33.4)	1298 (6.1)

*Sw, seawater; Mad M. decactis; MadBle bleached M. Decactis; #, number*.

## Discussion

Microbial assemblages during LLR (Sw14) were comparable to those previously described for the surface layers in the western SAO (South Atlantic Gyral - SATL) (Alves Junior et al., 2015) and within the WTRA (Heywood et al., 2006; Schattenhofer et al., 2009), where the dominant groups (=50%) detected were also *Prochlorales* and unclassified Alphaproteobacteria or SAR11 and related. Following LLR the microbial assemblages observed increasingly differed from previous studies focusing the surface layers of those most neighboring locations (Heywood et al., 2006; Schattenhofer et al., 2009; Swan et al., 2011). *Alteromonadales* appeared as the second most abundant group after *Prochlorales*, prevailing over unclassified Alphaproteobacteria, and *Vibrionales* emerged as a new group with >5% relative abundance (Sw15). Comparable *Alteromonadales* relative abundances, combined with lower abundances of unclassified Alphaproteobacteria (SAR11) were previously reported for the sub-superficial chlorophyll maximum (SCM) layer at higher depths (48–82 m) in the SAO, where, instead of *Vibrionales, Pseudomonadales*, and *Mamiellales* emerged as differing groups compared to the surface layers (Alves Junior et al., 2015). At HHR (Sw18), other Gammaproteobacteria appeared with > 5% relative abundance, i.e., *Pseudomonadales* and *Oceanospirillales*, whereas Thaumarchaota reached Euryarchaeota relative abundance levels. *Pseudomonadales, Oceanospirillales*, and Thaumarchaeota characterized deep waters (236–1200 m) in the SAO, and in which water masses *Prochlorales* was not amongst the 10 most abundant orders (AlvesJr-14). (Schattenhofer et al., 2009) reported a Gammaproteobacteria bloom in the North Atlantic Drift Province (NADR), with a maximum relative abundance of >50% of all picoplankton in surface waters, compared to the average values of 2–5% for all the other Atlantic provinces. Only a minor fraction was identified (*Alteromonas/Colwellia and Pseudoalteromonas: 2–5%; Vibrio: 1%, and Oceanospirillum:* 4%). The Gammaproteobacteria bloom was attributed to the end of the spring phytoplankton bloom, indicated by declining chlorophyll values. Massive growth of *Bacteroidetes* was concomitant and deep water Archaea presence at surface was not observed. Gammaproteobacteria have the potential to respond to sudden nutrient pulses released from phytoplankton (Cottrell and Kirchman, 2000). Members of *Alteromonas, Pseudoalteromonas*, and *Vibrio* are well known to rapidly respond to excess nutrient supply (Bano and Hollibaugh, 2002; Beardsley et al., 2003; Allers et al., 2007, 2008). Thaumarchaeota are typically more abundant at depths of ≥100 m, as oposed to Euryarchaeota, which is known for decreasing abundance below 100 m (Delong, 1992; Zhang et al., 2009; Santoro et al., 2010; Tseng et al., 2015). In SPSPA, concomitant with Thaumarchaeota increase, ammonia levels decreased, possibly due to its ammonia-oxidizing ability (Francis et al., 2005); and *Flavobacteriales* (*Bacteroidetes*) relative abundance decreased. Next (Sw22), the five most abundant groups at LLR recovered relative abundances almost to LLR (Sw14) levels (*Prochlorococcus*, unclassified Alphaproteobacteria—SAR11, *Rhodobacterales, Rhizobiales*, and *Chroococcales*), but *Alteromonadales, Pseudomonadales, Vibrionales*, and *Oceanospirillales* remained amongst the 10 most abundant groups, as well as Thaumarchaeota remained abundant, which is a distinctive assemblage for those geographical coordinates (Schattenhofer et al., 2009; Swan et al., 2011; Alves Junior et al., 2015). Shifts in planktonic assemblages at the mesophotic zone in SPSPA were possibly driven by the turbulence surge, meaning that microbes from progressively deeper layers could hitchhike with the vertical flux along the surge. The upwelling is also supported by the enrichment and by the wide variation of water temperatures registered for the sampling depth (Supplementary Figure 4).

### A model of physical-chemical-biological dynamics in SPSPA

**Figure 4:**
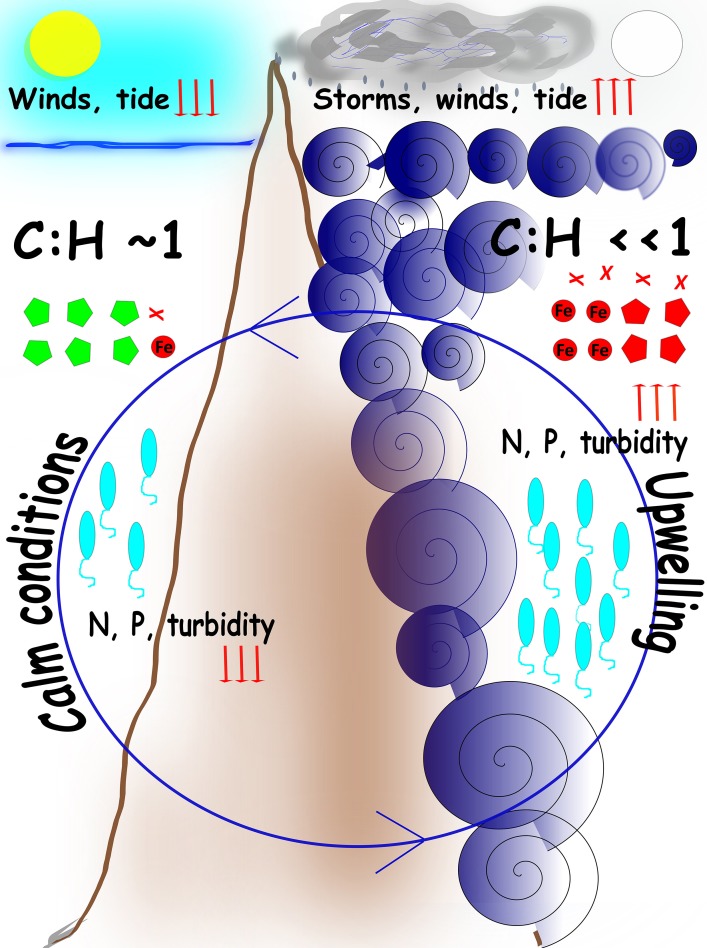
**Cartoon model of the main processes in SPSPA Cyclic turbulence-nutrient pulses determine transient shifts in the bacterioplankton**. Circular central blue arrow indicates the cyclic nature of the events. **Left panel:** during LLR calm tides, weak winds, absence of clouds, and clear waters correlate with lower levels (downwards red arrows) of nitrogen (N) and phosphorous (P), and equivalence between autotrophy and heterotropy, represented by the ratio cyanobacteria:heterotrophs (C:H) equalizing 1. Cyanobacterial phages (green poligons) reflect the abundance of the hosts. **Right panel:** during HHR harsh conditions are determined by violent turbulence resulting in upwelling and enrichment, represented by high levels (upwards red arrows) of N and P, and a shift to heterotrophy dominance, represented by a low ratio cyanobacteria:heterotrophs (C:H < < 1). Intense winds, cloudiness and rain, that washes the rocks covered with guano, contribute to enrichment and the shift to heterotrophy. Proteobacterial phages (red poligons) reflect the abundance of the hosts as well as Iron acquisition (red circle) and virulence factor (red crosses) genes. Vibrios abundance (blue elipses) reflect seawater parameters. Episodic surges frequently correlate with full moon. When turbulence pulses mitigate and weather assuages a recovery takes place in seawater, both in terms of nutrients concentrations and microbial assemblages (e.g., after moon changes toward new).

Although sequence similarities to genes do not represent levels of gene expression, metagenomes have been shown to be strong predictors of the biogeochemical conditions driving the microbial community (Dinsdale et al., 2008). According to the lines of evidence garnered the microbiome of the mesophotic waters in SPSPA undergoes cyclic transient shifts in relation to turbulence-nutrients regimes. A microbial succession resulting from the interplay between physical and chemical factors is a plausible scenario. Two extreme turbulence-nutrient regimes can be clearly distinguished and alternate with intermediate conditions determining microbial assemblages: (i) When turbulence is low (LLR) at least 50% of the microbiome is composed of *Prochlorococcus*, followed by unclassified Alphaproteobacteria (SAR11 and related), which are small sized cells, highly adapted to oligotrophic conditions and starvation. *Rhodobacterlaes, Rhizobiales*, and *Chroococcales* are typical. In this environment phage genes are the most abundant in seawater, mostly from *Prochlorococcus* and *Synechococcus*, following the hosts' abundances. The viral shunt is probably less active toward relatively scarce cells. The proportion of unknown genes is the highest; (ii) Episodic surges promote vertical mixing from the immediate lower water mass to the mixed layer. Waves also wash guano from the cays flushing phosphates and ammonium into the inlet. Heterotrophs (*Alteromonadales, Vibrionales*) respond quickly and surpass autotrophs, motility, and chemotaxis related genes stand out; (iii) Ongoing eddies and intensified high-energy waves promote entrainment of deep water organisms such as Thaumarchaeota and nutrients (nitrite, nitrate) (HHR). Eventual cloudiness, winds and rain cope with turbulence, irradiance is intermittent and turbidity is enhanced. Heterotrophy predominates with dominance of *Alteromonadales, Pseudomonadales*, and *Oceanospirillales*. Gammaproteobacterial groups approximate 50% of the microbial assemblage, resembling the end of the spring phytoplankton bloom in higher latitudes (e.g., NADR). The gene pool in surface waters reflects the shift with membrane transport and virulence-related genes (e.g., TonB-dependent receptor, *V. cholerae* virulence genes) surpassing cyanobacterial phages and basic metabolisms genes. Phages targeting heterotrophs are active. The proportion of unknown genes is the lowest; (iv) Turbulence alleviates (e.g., after moon changes toward new). Larger cell sized heterotrophs begin to decline as viral lysis and predation by grazers overrides growth, which is constrained by the paucity of limiting nutrients (e.g., phosphorus). The microbial loop is most prominent at this stage. Autotrophs respond to irradiance and retake growth (if rain, wind, and cloudiness mitigate this response is accelerated). Nitrogen metabolism is intensive. A reversal to autotrophy:heterotrophy equilibrium is triggered (Figure 4). The short-lived but recurrent turbulence-nutrient pulses might be responsible for structuring the marine ecosystem in a bottom-up manner in SPSPA. These pulses might be indispensable to warrant the energy and carbon flow to the higher trophic levels concurring to the observed pelagic fishes biomass around the barren islets (Luiz and Edwards, 2011). On a stable LLR the growth of phytoplankton is largely supported by regenerated nutrients, so only a small proportion of primary production is available to higher trophic levels or for export to the deep sea (Cullen et al., 2002; Karl, 2014). Turbulence is physically forcing the co-ocurrence of nutrients and light in SPSPA, on the other hand, the fact that nutrient resupply is short-lived might concur to retain the local mesotrophic condition. Bacterioplankton shifts were shown to be transient, following the cyclic nutrient-turbulence pulses and other physical parameters (rain, cloudiness, winds, turbidity).

### The holobiont *madracis decactis*

Some bacterial taxa prevalent in bleached corals (*Rhodobacterales, Rhizobiales*, and *Clostridiales*) have been previously associated with opportunistic diseases (Frias-Lopez et al., 2002; Rosenberg et al., 2007; Sekar et al., 2008; Sunagawa et al., 2009; Mouchka et al., 2010). On the other hand, a study of the corals microbiome, aiming at distinguishing the core, the symbiotic and the whole community microbiome, suggested that *Rhodobacterales* pertains to the latter. Conversely, *Rhizobiales* members were suggested to belong to the symbiotic coral microbiome. *Actinomycetales* and *Burkholderiales* (both also prevalent in all coral samples) were characterized as part of the coral core microbiome (D Ainsworth et al., 2015). *Pasteurellales* was one of the most abundant bacteria in the coral metagenomes, contrasting to its dwindling relative abundance in seawater. *Pasteurellales* members can cause disease in a wide range of domestic and wild animals (Wilson and Ho, 2013). They are commonly found in fish tissues (Birkbeck et al., 2002). Reef fishes (Chaetodontidae) have been characterized as major vectors of coral diseases (Raymundo et al., 2009) and damselfish (*Stegastes* spp.) was shown to increase the prevalence of the coral Black Band Disease (BBD) (Casey et al., 2014). It is plausible that *Pasteurellales* are frequently transmitted to corals through fishes, possibly by fish bites, since *M. decactis* is frequently predated by *S. sanctipauli, H. radiatus* and other fishes in SPSPA (Supplementary Figure 3). This hypothesis explains the uneven distribution of *Pasteurellales* between healthy and diseased corals, as well as the disconnection to seawater parameters. Bleached corals were distinguished by enhanced Iron Acquisition metabolism. Six functions within this subsystem were represented only in bleached corals. HHR bleached corals samples (MadBle18) were the most dissimilar in terms of sequence composition (di- and tetranucletides frequencies), higher counts of Proteobacteria (including *Vibrionales*), and higher relative abundance of hits to the VFDB, including *V. cholerae*-related VFs. This dissimilarity, including the HHR healthy coral (Mad18), indicates that the healthy coral holobiont might be less sensitive to transient seawater-related perturbations than the diseased animals. The distinguishing characteristics of HHR bleached corals agree with the bacterioplankton and seawater features during HHR, reported both in the present and former study (Supplementary Figure 4; Moreira et al., 2014): sequence composition, higher relative abundance of motility and chemotaxis, and of membrane transport and virulence genes (e.g., Ton-B dependent receptor of the Iron Acquisition metabolism, *V. cholerae*-related VFs), higher vibrio counts and nutrients in seawater. Taken together, the datasets suggest coupling between the benthic and pelagic compartments, as previously reported (Chimetto Tonon et al., 2015).

### Caveats

Owing to the remote nature of this site, we do not have complete data sets. Resampling will be needed to strengthen the link between turbulence-upwelling and the shifts in microbial assemblages.

## Conclusions

This work analyzed shifts in microbial composition related to physical forcings (turbulence-upwelling and storms) in SPSPA. LLR is characterized by the equilibrium between autotrophy-heterotrophy and microbial assemblages that resemble those of surface tropical waters previously characterized in the SAO. At HHR microbial communities shift to heterotrophic and deep-sea characteristic organisms (Thaumarchaota). HHR diseased corals are distinguished by sequence composition and enhanced VFs hits, suggesting some level of coupling between planktonic and coral microbial communities.

### Conflict of interest statement

The authors declare that the research was conducted in the absence of any commercial or financial relationships that could be construed as a potential conflict of interest.

## Abbreviations

EUC: Atlantic Equatorial Undercurrent
BLAST: Basic Local Alignment Search Tool
chla: chlorophyll a
CFU: colony forming unit
DMSP: dimethylsulfoniopropionate
HOT: Hawaii Ocean Time-series
HHR: high turbulence-high nutrients regime
LLR: low turbulence-low nutrients regime
MAR: Mid-Atlantic Ridge
NCBI: National Center for Biotechnology Information
NADR: North Atlantic Drift Province
PHAST: PHAge Search Tool
SeaWiFS: Sea-viewing Wide Field-of-view Sensor
SATL: South Atlantic Gyral
SAO: South Atlantic Ocean
SPSPA: St Peter and St Paul Archipelago
FZ: St Paul Fault Zone
SEC: South Equatorial Current
SCM: sub-superficial chlorophyll maximum
VFs: virulence factors
VFDB: Virulence Factor Data Base
WTRA: Western Tropical Atlantic Province
WORMS: World Register of marine Species.
