# Supplementary material for: Turbulence-driven shifts in holobionts and planktonic microbial assemblages in St. Peter and St. Paul Archipelago, Mid-Atlantic Ridge, Brazil
Source: Front Microbiol. 2015 Oct 2;6:1038. doi: 10.3389/fmicb.2015.01038 (PMC4591530; doi:10.3389/fmicb.2015.01038)
Supplement: Supplementary file 3 [file Presentation1.PDF]

Supplementary Material

Turbulence-driven shifts in holobionts and planktonic microbial assemblages in St Peter and St Paul Archipelago, Mid-Atlantic Ridge, Brazil

Ana Paula B. Moreira[1], Pedro M. Meirelles[1], Eidy de O. Santos[2], Gilberto M. Amado-Filho[3], Ronaldo Bastos Francini-Filho[4], Cristiane C. Thompson[1], Fabiano L. Thompson[1*]

[1]Laboratory of Microbiology, Institute of Biology, Federal University of Rio de Janeiro (UFRJ), Rio de Janeiro, Brazil
[2]Fundação Centro Universitário Estadual da Zona Oeste (Uezo), Rio de Janeiro, Brazil
[3]Diretoria de Pesquisa Científica, Instituto de Pesquisas Jardim Botânico do Rio de Janeiro (JBRJ), Rio de Janeiro, Brazil
[4]Department of Environment and Engineering, Federal University of Paraíba (UFPB); Rio Tinto, Brazil.

*** Correspondence:** Av. Carlos Chagas Fº. S/N – CCS – IB – BIOMAR – Laboratório de Microbiologia – Bloco A (Anexo) A3 – sl 102 – Cidade Universitária, Rio de Janeiro – RJ, Brasil. CEP 21941-599. Telephone/fax: 55 21 3938-6567; Email: fabianothompson1@gmail.com

1. Supplementary Table

Supplementary Table 1 The most abundant genes per metagenome (provided as supplemental .XLSX file)

2. Supplementary Video

Supplementary Video 1 Surge events in St Peter and St Paul Archipelago along days 19-21/Sep/2010 (provided as supplemental .MP4 file) (Video: Ana Paula B. Moreira)

3. Supplementary Figures

Supplementary Figure 1. Study area. **A** Location of St Peter & St Paul Archipelago (SPSPA). Map overlayied with chlorophyll a (chla) image from the Sea-viewing Wide Field-of-view Sensor (SeaWiFS). **B** Enlargement of the view to detach the chla pattern. SeaWiFs local average value for eight days 14-21/Sep/2010 is highlighted: 0,15538 mg/m^3. **C** Satellite (SIO NOAA, US Navy, NGA GEBCO) view of SPSPA showing the U-shaped inlet encompassed by the islets (Belmonte, São Pedro, São Paulo and Barão de Teffé), the Scientifique Station (SS) and coordinates (00°55'N; 29°22'W). Scale bar is 49m. **D** View of the inlet from the SS, at the low turbulence-low nutrients regime (LLR) (photo: Ana Paula.B. Moreira).

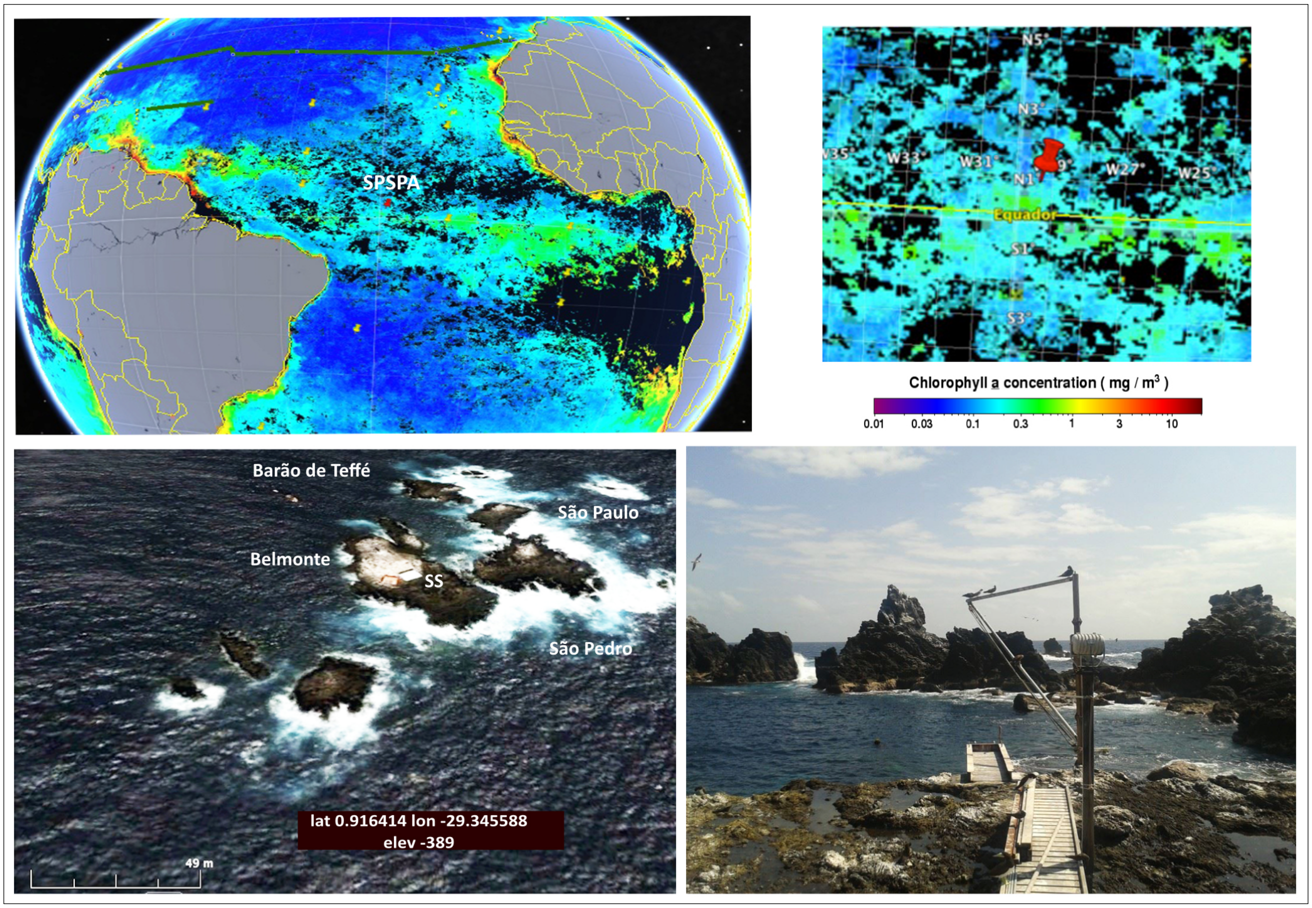

Supplementary Figure 2. **Geomorphology.** **A** Mid-Atlantic Ridge (MAR) on the St Paul's Fault Zone (FZ) at SPSPA coordinates. **B** Topography at the FZ where SPSPA stands, with the profile of the inlet inside which sampling was performed. Images generated with GeoMapApp© available at http://www.geomapapp.org (Ryan et al.,2009). **C** Depht color scale.

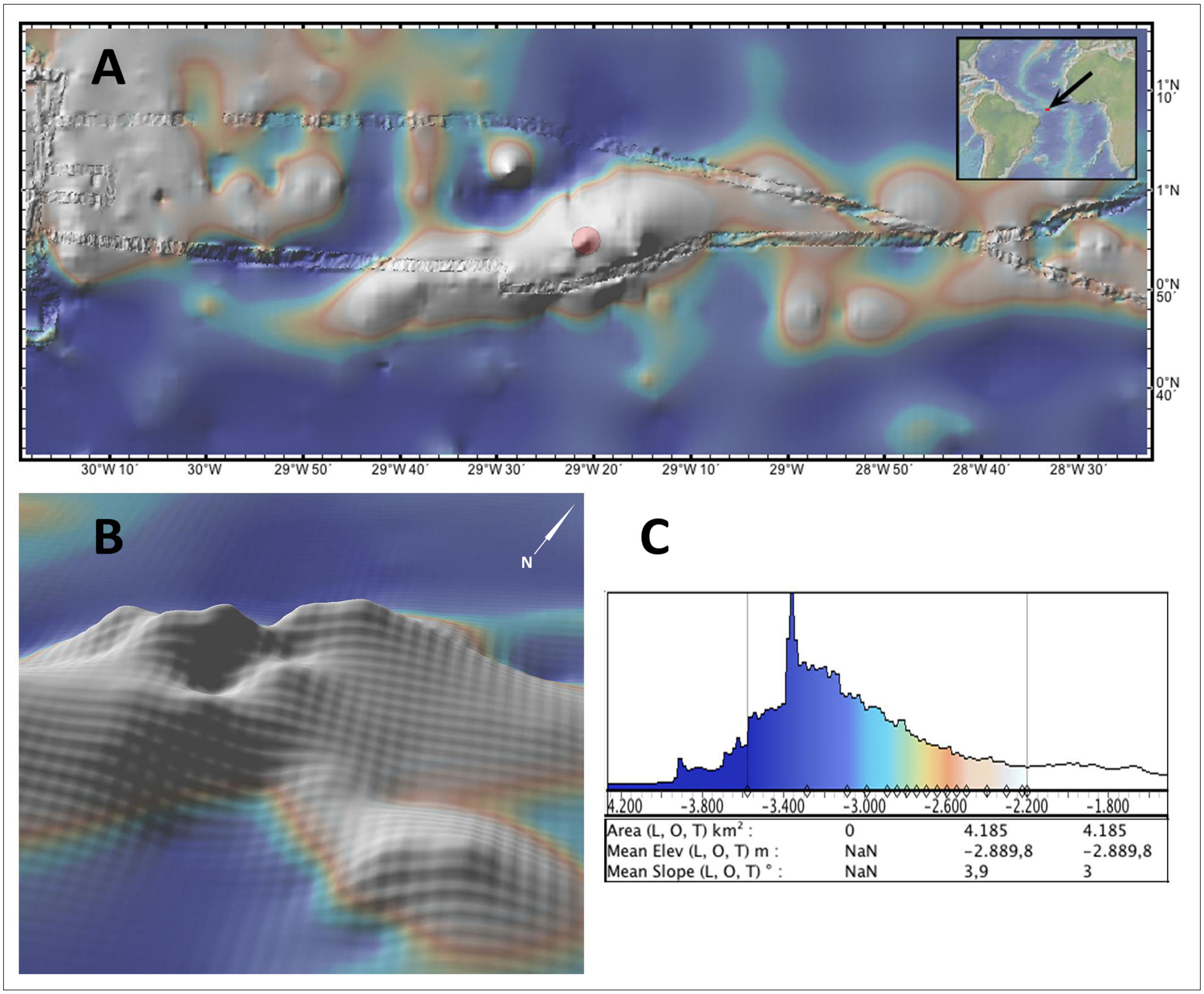

Supplementary Figure 3. ***Madracis decactis*** **in SPSPA. A** Healthy **B** Bleached **C-D** With scars left by *Stegastes sanctipauli* and *Halichoeres radiatus* predation, respectively (photos: Ronaldo B. Francini-Filho).

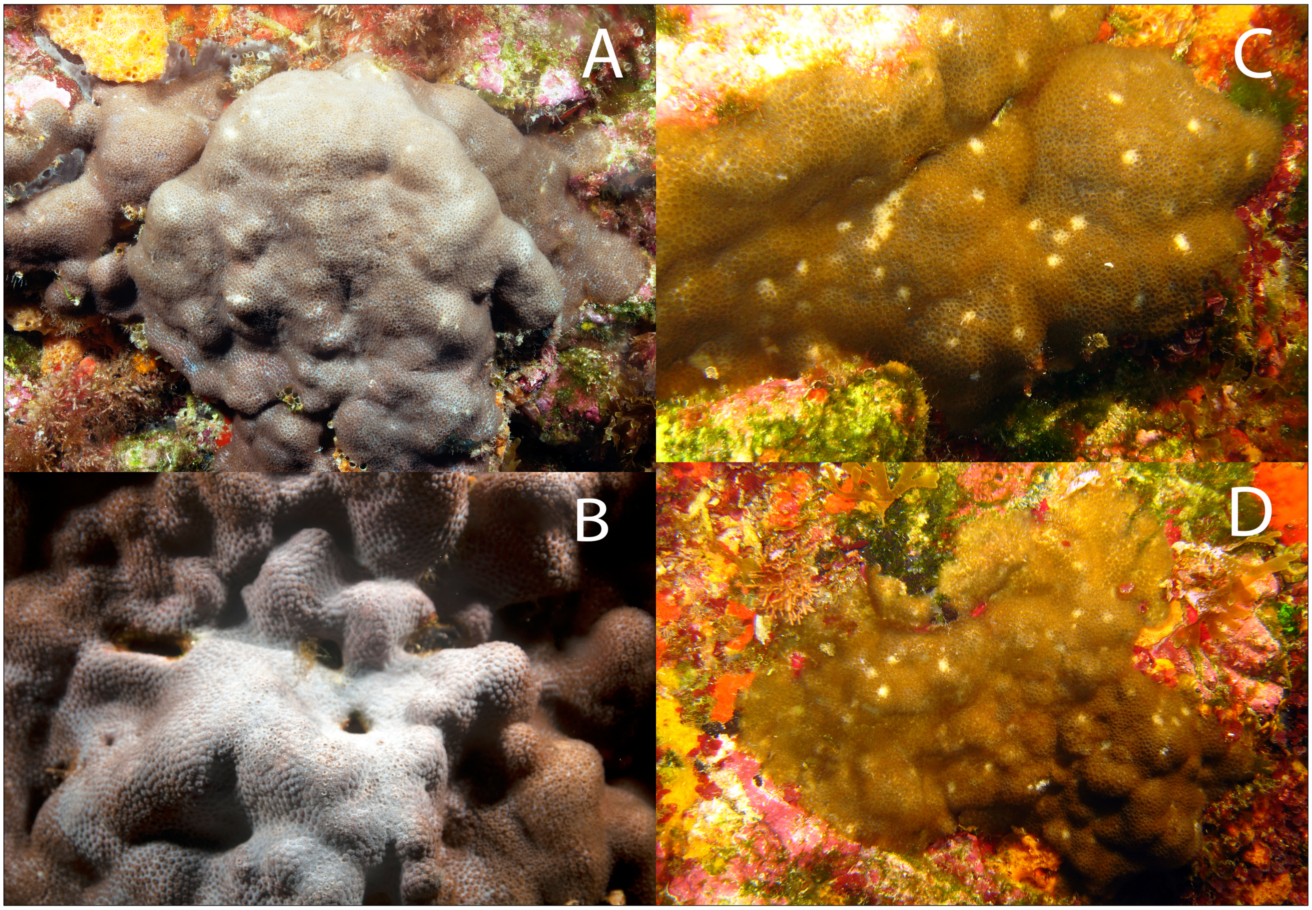

Supplementary Figure 4. Environmental parameters A Nutrients and vibrio counts in seawater. Pooled data from Moreira et al. (14). Scale bar for nutrients at left, except for Total nitrogen, whose scale is shown at the right side of the graph. Both scales are μM.L^{-1}. Vibrios counts are expressed as colony forming units, CFU.mL^{-1}. **B** Temperature-depht profile from Crespo et al. (14). Measures were done in Sep/2010 and Jun/2011, five replicates each. Bars represent standard deviation for the average.

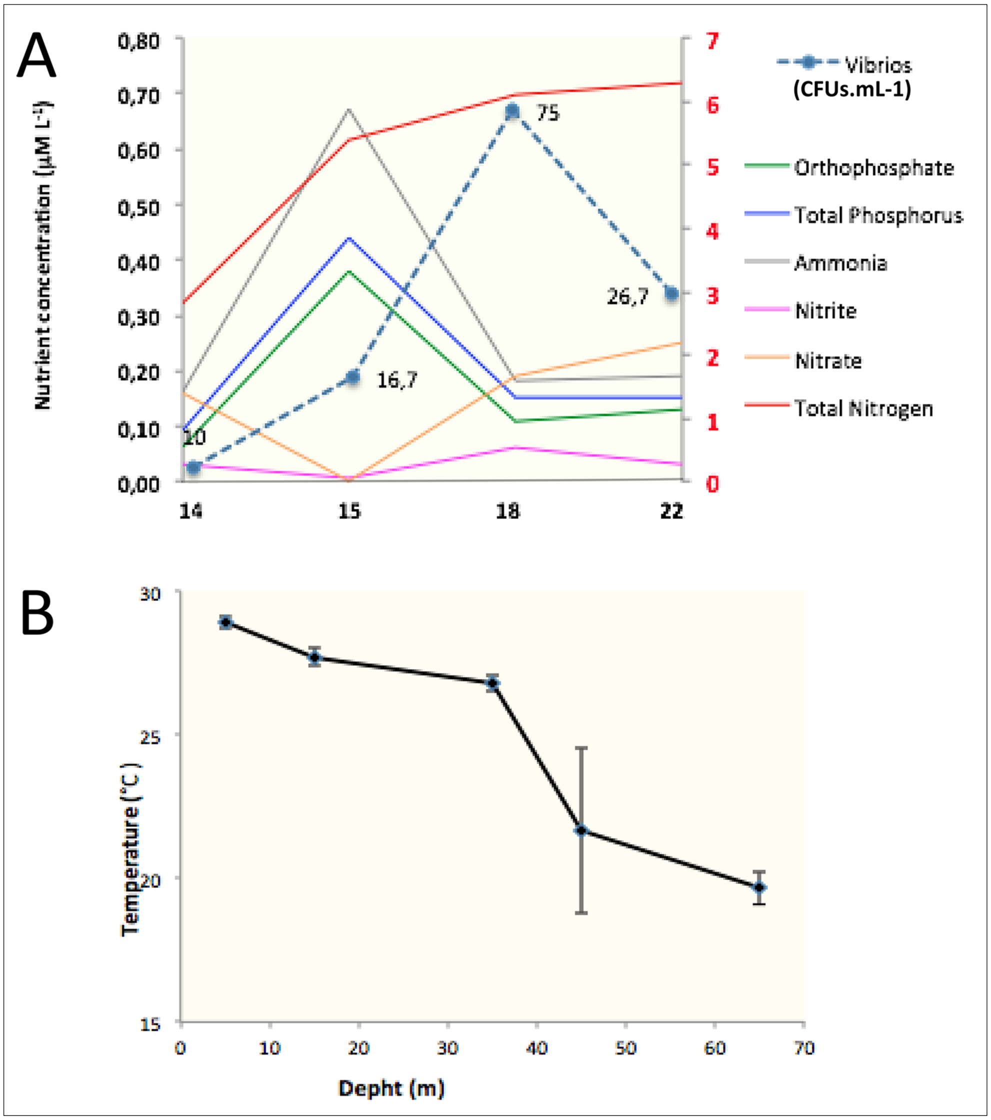

Supplementary Figure 5. Karlin disctances (δ) for seawater metagenomes. Karlin matrix with the dinucleotides dissimilarities for all pairs of seawater metagenomes (multiplied by a 1,000). Average δ values between pooled replicates are shown at bottom left and δ categories of genetic similarity defined by δ ranges are shown at bottom right. Sw, seawater.

Sample	Sw14-3	Sw14-1	Sw14-2	Sw15-4	Sw15-1	Sw15-2	Sw18-3	Sw18-1	Sw18-2	Sw22-2	Sw22-3	Sw22-1
Sw14-3	0	11,5	7,3	36,6	24,2	21	48,1	51,2	61,4	17,3	25,1	26,4
Sw14-1		0	7,6	29,9	14,5	11,6	42,1	44,1	54	8,8	17	16,7
Sw14-2			0	32,8	18	17	46,7	48,8	58,7	13,5	21	21,1
Sw15-4				0	17,7	23,7	25,8	24,4	31,7	22,8	16,3	14,3
Sw15-1					0	7,8	31,7	33,8	43,7	8,6	9,9	5,1
Sw15-2						0	36	38	48,7	5,7	10,9	10,1
Sw18-3							0	9,8	19,6	35,1	26,4	26,7
Sw18-1								0	14	37,2	27,8	28,7
Sw18-2									0	47,3	38,7	38,7
Sw22-2										0	9,4	9,4
Sw22-3											0	5,6
Sw22-1												0

	Average δ		δ Categories	
Sw14-Sw14	8,8	<11	11< δ < 30	>30
Sw-14-Sw15	22,9	Sw14-Sw14	Sw15-Sw15	Sw-14-Sw18
Sw-14-Sw18	50,6	Sw22-Sw22	Sw18-Sw18	Sw15-Sw18
Sw14-Sw22	18,5		Sw-14-Sw15	Sw18-Sw22
Sw15-Sw15	16,4		Sw14-Sw22	
Sw15-Sw18	34,9		Sw15-Sw22	
Sw15-Sw22	11,5			
Sw18-Sw18	14,4			
Sw18-Sw22	34,1			
Sw22-Sw22	8,1			

Supplementary Figure 6. Karlin disctances (δ) for *M. decactis* metagenomes. Karlin matrix with the dinucleotides dissimilarities for all pairs of *M. decactis* metagenomes (multiplied by a 1,000). Average δ values between pooled replicates are shown at bottom left and δ categories of genetic similarity defined by δ ranges are shown at bottom right. Mad, healthy *M. decactis;* MadBle, bleached *M. decactis.*

Samples	Mad14	Mad15-1	Mad15-2	Mad18	MadBle18-1	MadBle18-2	MadBle22-1	MadBle22-2	Mad22
Mad14	0								
Mad15-1	7,4	0							
Mad15-2	12,9	18,7	0						
Mad18	9,6	12,4	14,3	0					
MadBle18-1	29,4	36,9	18,6	32,5	0				
MadBle18-2	44,6	51,3	33,6	46,8	15,4	0			
MadBle22-1	8,2	4,6	19	10,4	37,3	51,5	0		
MadBle22-2	4,2	8,3	12,7	10,4	29,4	44,3	8,6	0	
Mad22	4,6	12	8,7	9,2	25,2	40,3	12,7	7	0

	Average δ
14-22	5,7
22-22	9,4
14-15	10,15
15-22	10,9
15-15	18,7
14-18	27,9
15-18	27,9
18-22	28,7
18-18	31,6

δ Categories

δ < 11	11< δ < 20	δ > 20
14-22	15-15	14-18
14-15		15-18
15-22		18-22
22-22		18-18

Supplementary Figure 7. Heatmaps reflecting the metagenomic tetranucleotides frequencies dissimilarities. A Heatmap reflecting seawater (Sw) metagenomic tetranucleotides frequencies dissimilarities. Samples Sw18 (high turbulence-high nutrients, HHR) were the most dissimilar to the remainders. The exception was sample Sw15-4, which clustered with samples Sw18. This pattern is the same of that obtained for the cluster dendogram (Fig. 1), in which sample Sw15-4 clustered with the Sw18 branch. **B** Heatmap reflecting corals metagenomic tetranucleotides frequencies dissimilarities. HHR bleached coral samples MadBle18 were the most dissimilar to all the remainders, including bleached coral samples MadBle22.

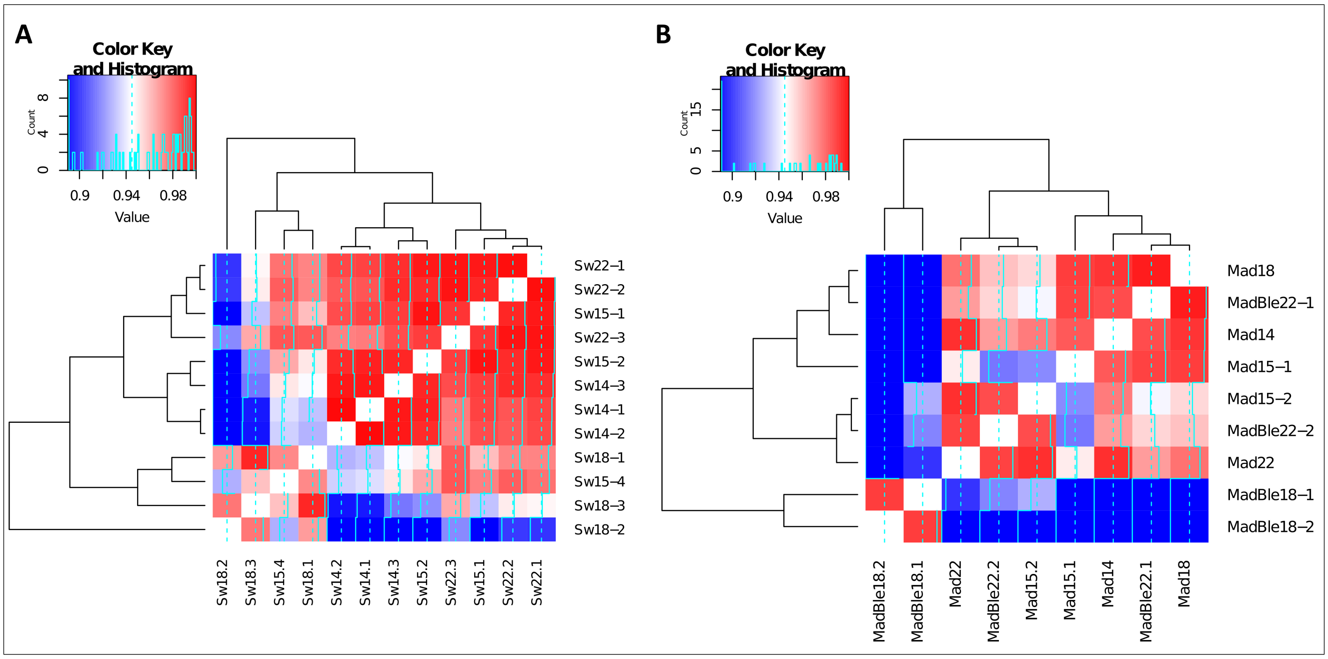

Supplementary Figure 8. Eukarya sequences in seawater and *M. decactis* metagenomes. Seawater samples Sw14 and 15, and Sw18 and 22 were pooled and indicated as Sw(14-15) and Sw(18-22), respectively. Healthy *M. decactis* samples Mad14 and 15, and Mad18 and 22 were pooled and indicated as Healthy Coral (14-15) and (18-22), respectively. Bleached *M. decactis* samples MadBle18 and 22 were pooled and indicated as Diseased Coral (18-22).

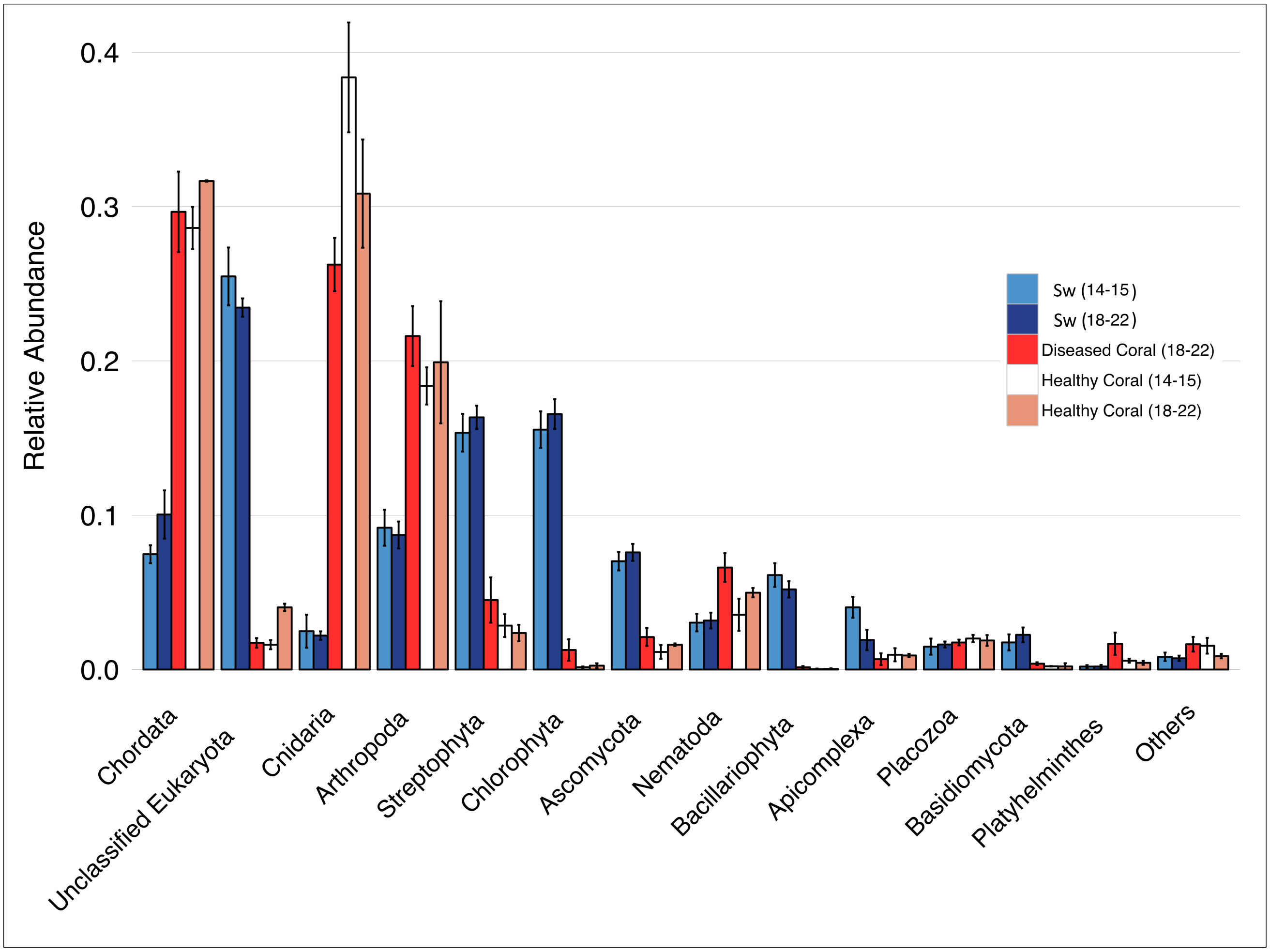

Supplementary Figure 9. Relative abundance of Proteobacteria in seawater metagenomes. Distribution of Proteobacteria classes in seawater samples with pooled replicates: Sw14 (1,2,3), -15 (1,2,4), -18 (1,2,3) and -22 (1,2,3). Regression lines for the Gamma- and AlphaProteobacteria relative abundances variation are shown at top by *red* and *orange* curves, respectively.

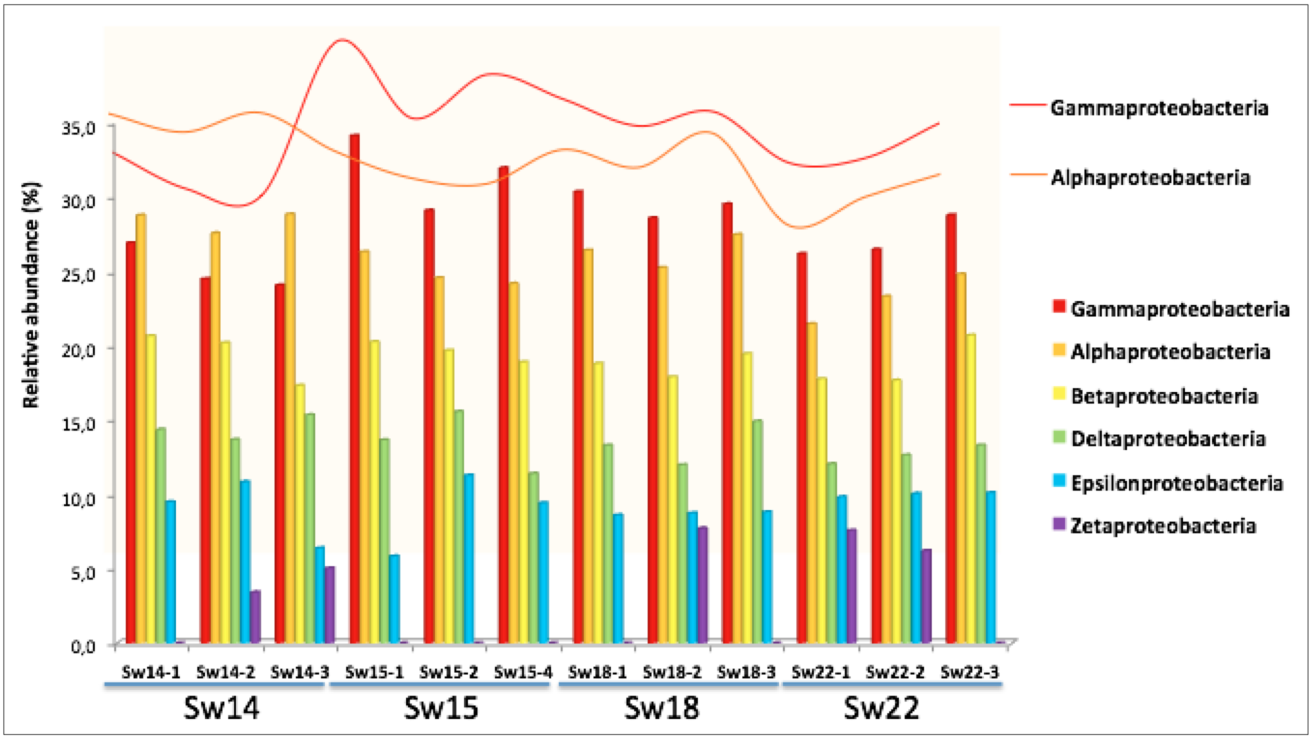

Supplementary Figure 10. Relative abundance of Subsystems in seawater metagenomes. Distribution of level 1 SEED subsystems is seawater metagenomes. All replicates are shown. Seawater 14-1, -2, -3 (Sw14, *green*), Seawater 15-1, -2, -4 (Sw15, *blue*), Seawater 18-1, -2, -3 (Sw18, *red*), Seawater 22-1, -2, -3 (Sw22, *yellow*).

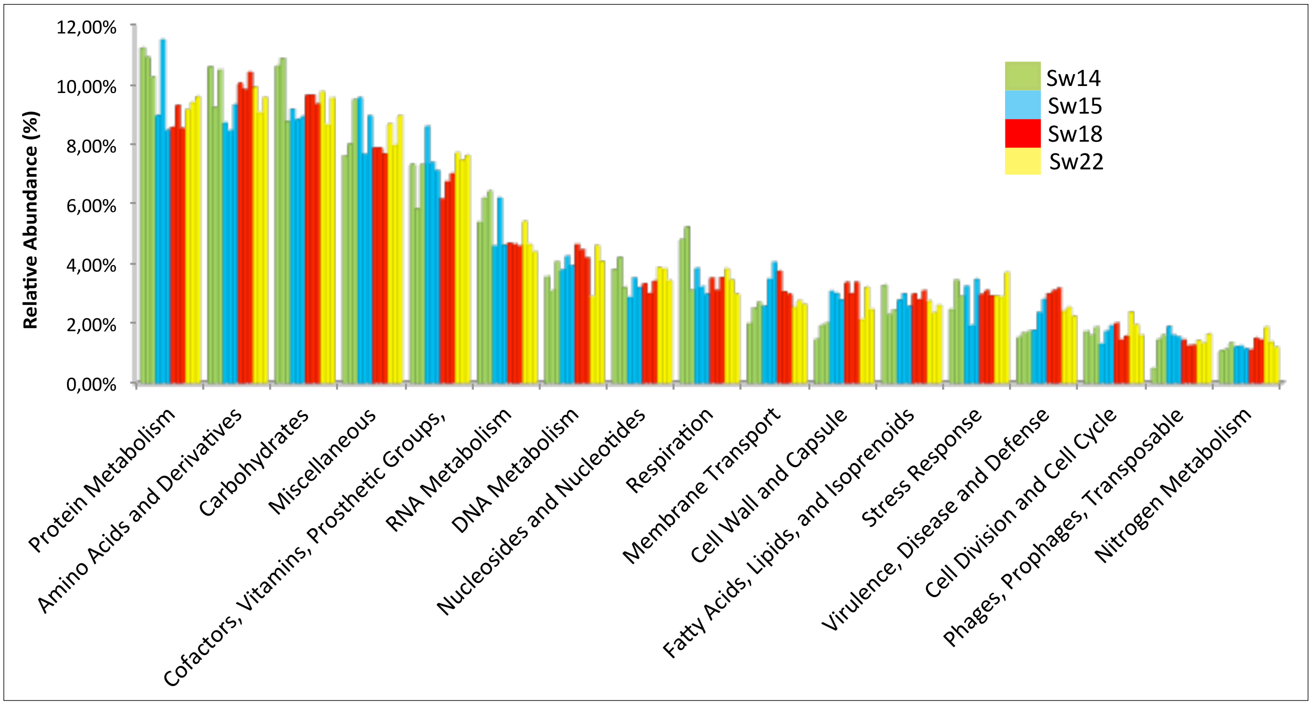

Supplementary Figure 11. Scheme of PHAGE_Synech_S_CRM01_NC_015569. Possible *Synechococcus* phage detected in seawater sample Sw14-1.

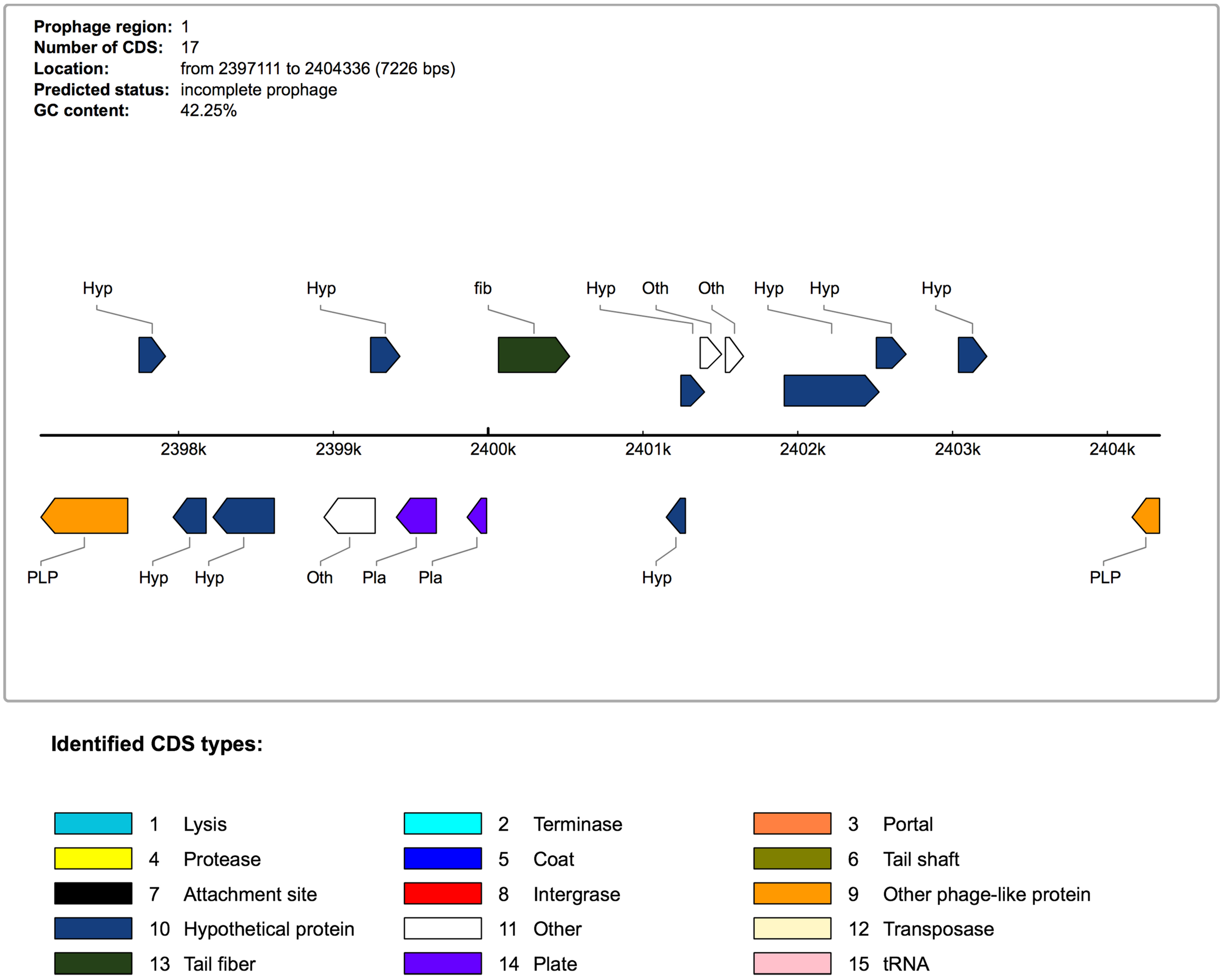

Supplementary Figure 12. Scheme of PHAGE_Prochl_P_SSM3_NC_021559. Possible *Prochlorococcus* phage detected in seawater sample Sw14-2.

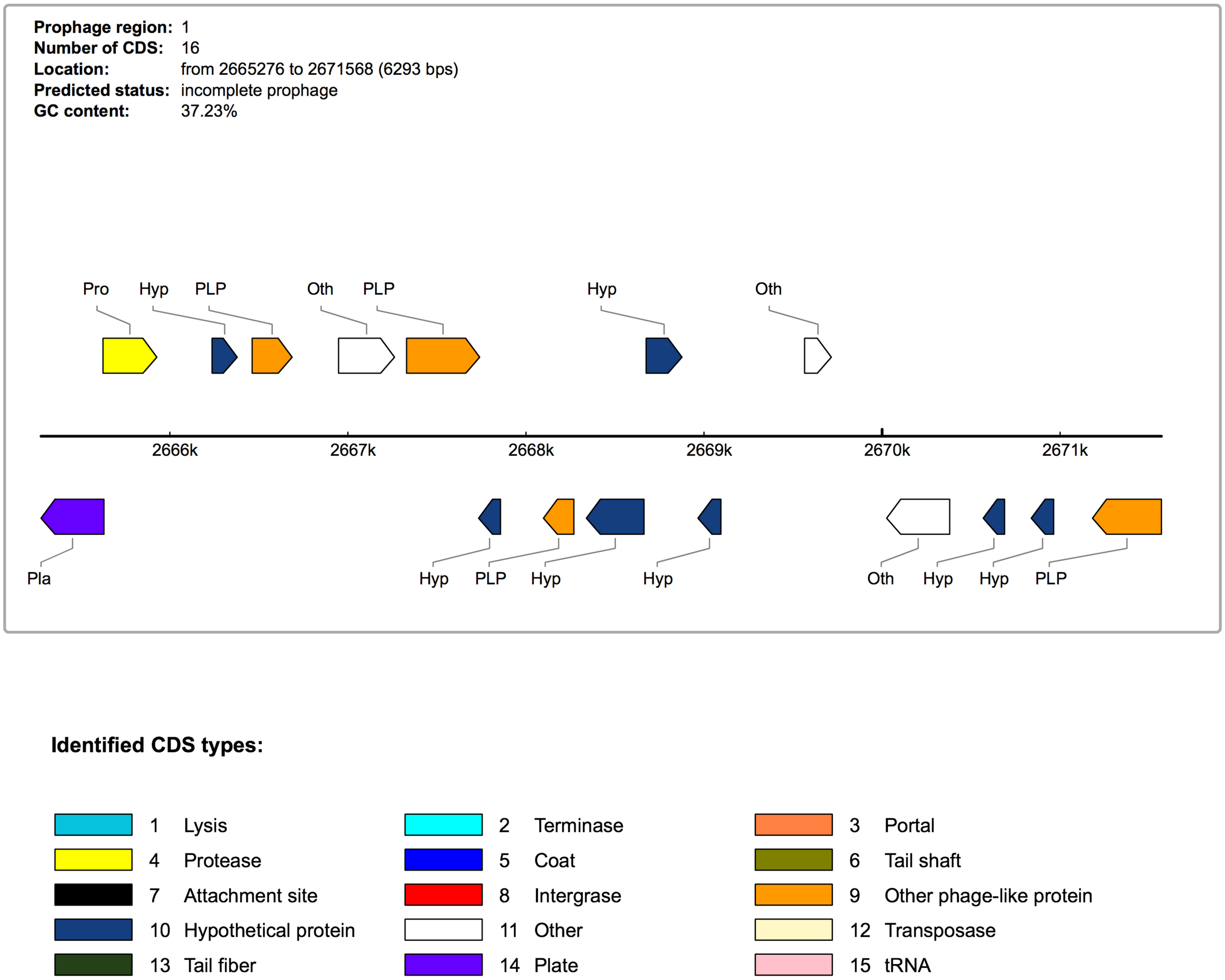

Supplementary Figure 13. Scheme of PHAGE_Aeromo_Aes012_NC_020879. Possible *Aeromonas* phage detected in seawater sample Sw14-3.

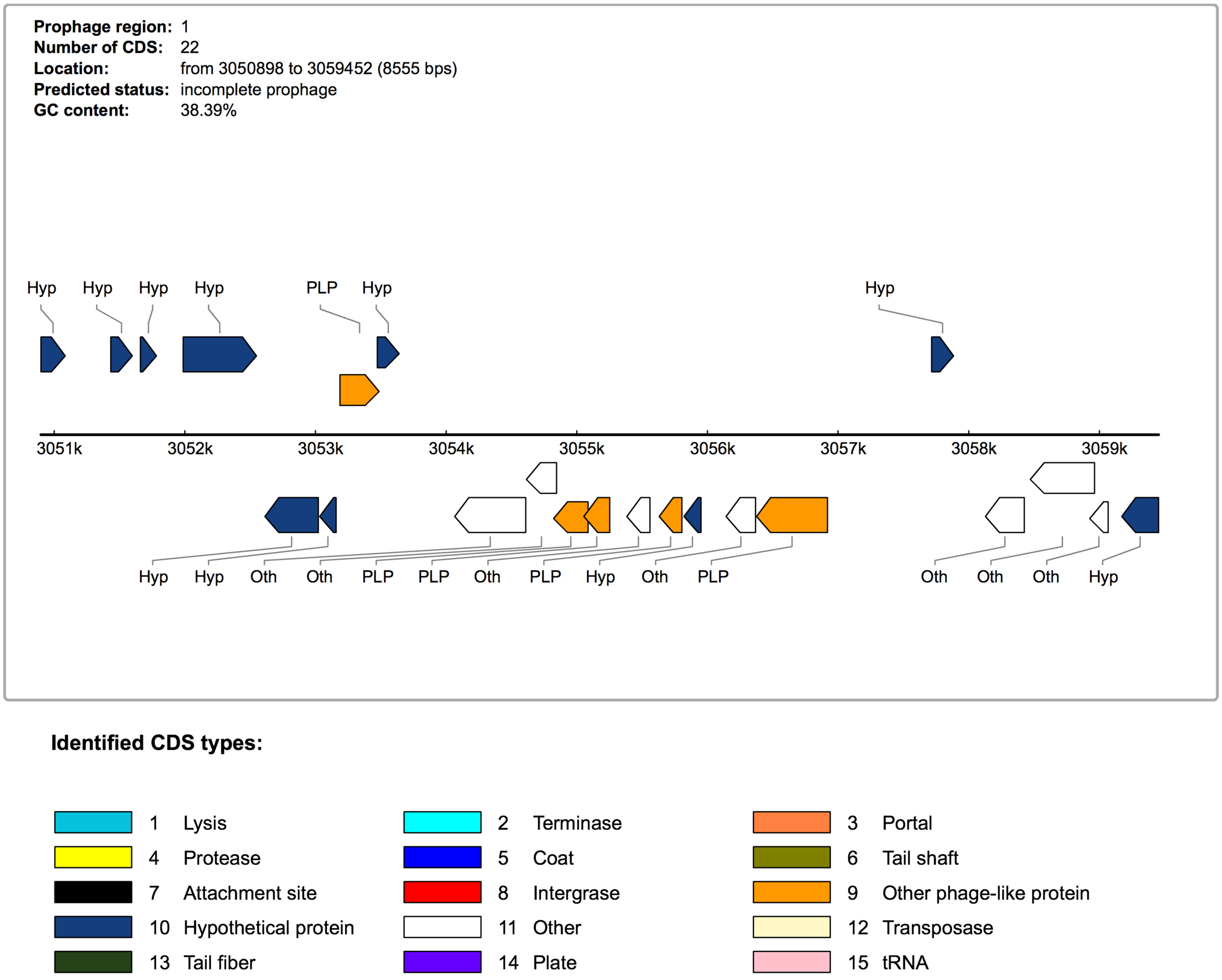

Supplementary Figure 14. Scheme of PHAGE_Bacill_G_NC_023719. Possible *Bacillus* phage detected in seawater sample Sw15-1**.**

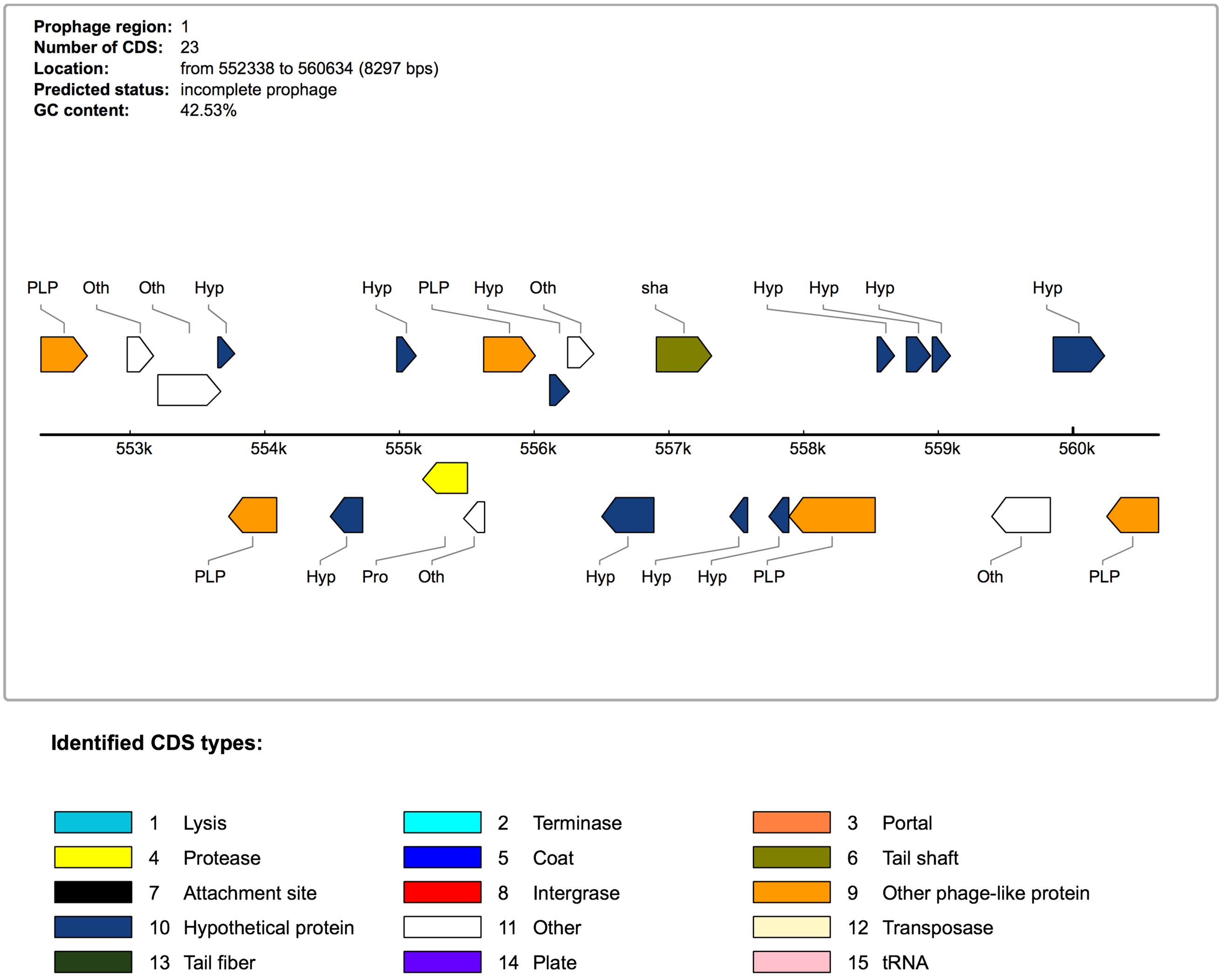

Supplementary Figure 15. Scheme of PHAGE_Ectoca_siliculosus_virus_1_NC_002687. Possible *Ectocarpus siliculosus* phage detected in seawater sample Sw15-2.

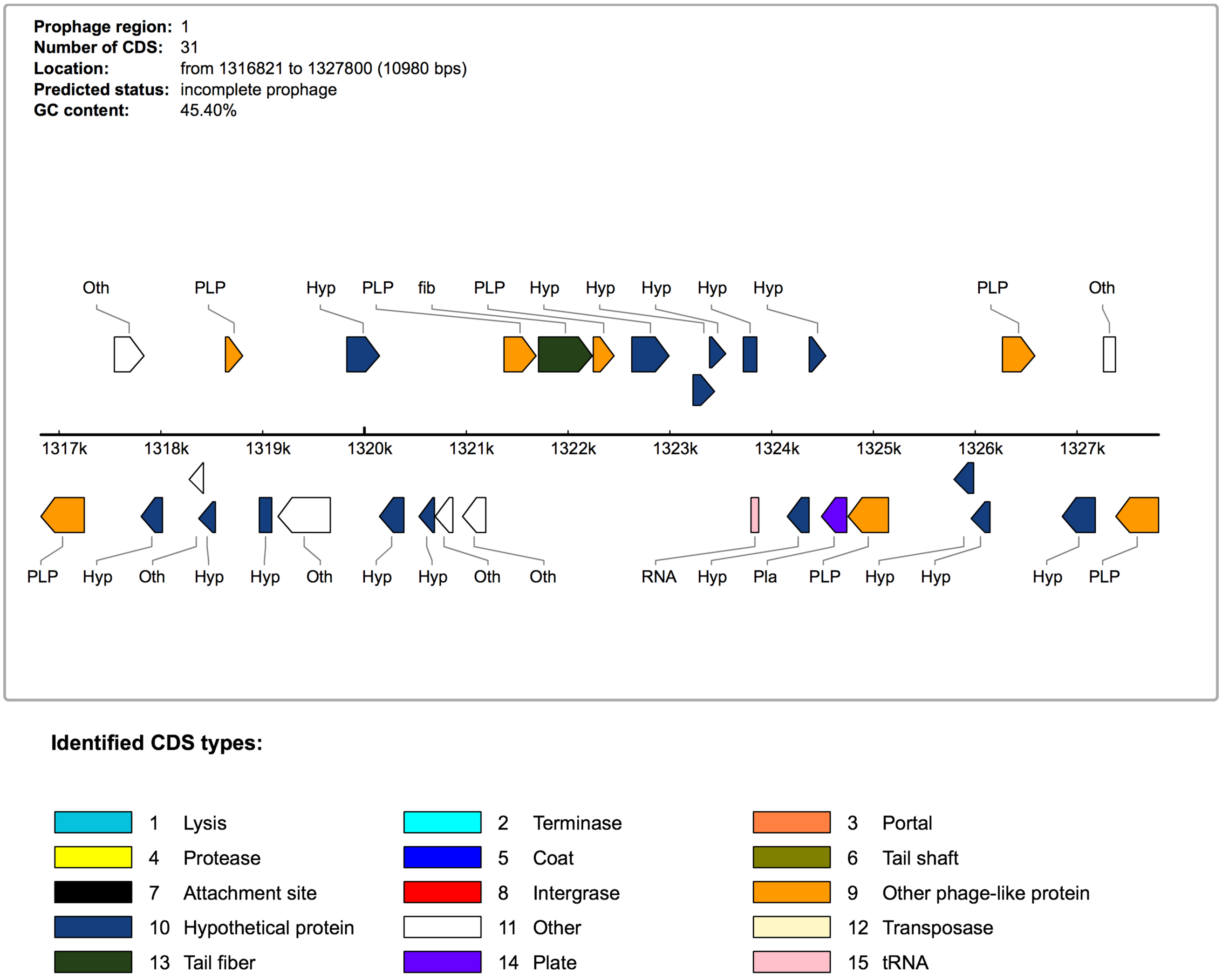

Supplementary Figure 16. Scheme of PHAGE_Bacill_G_NC_023719. Possible *Bacillus* phage detected in seawater sample Sw18-2**.**

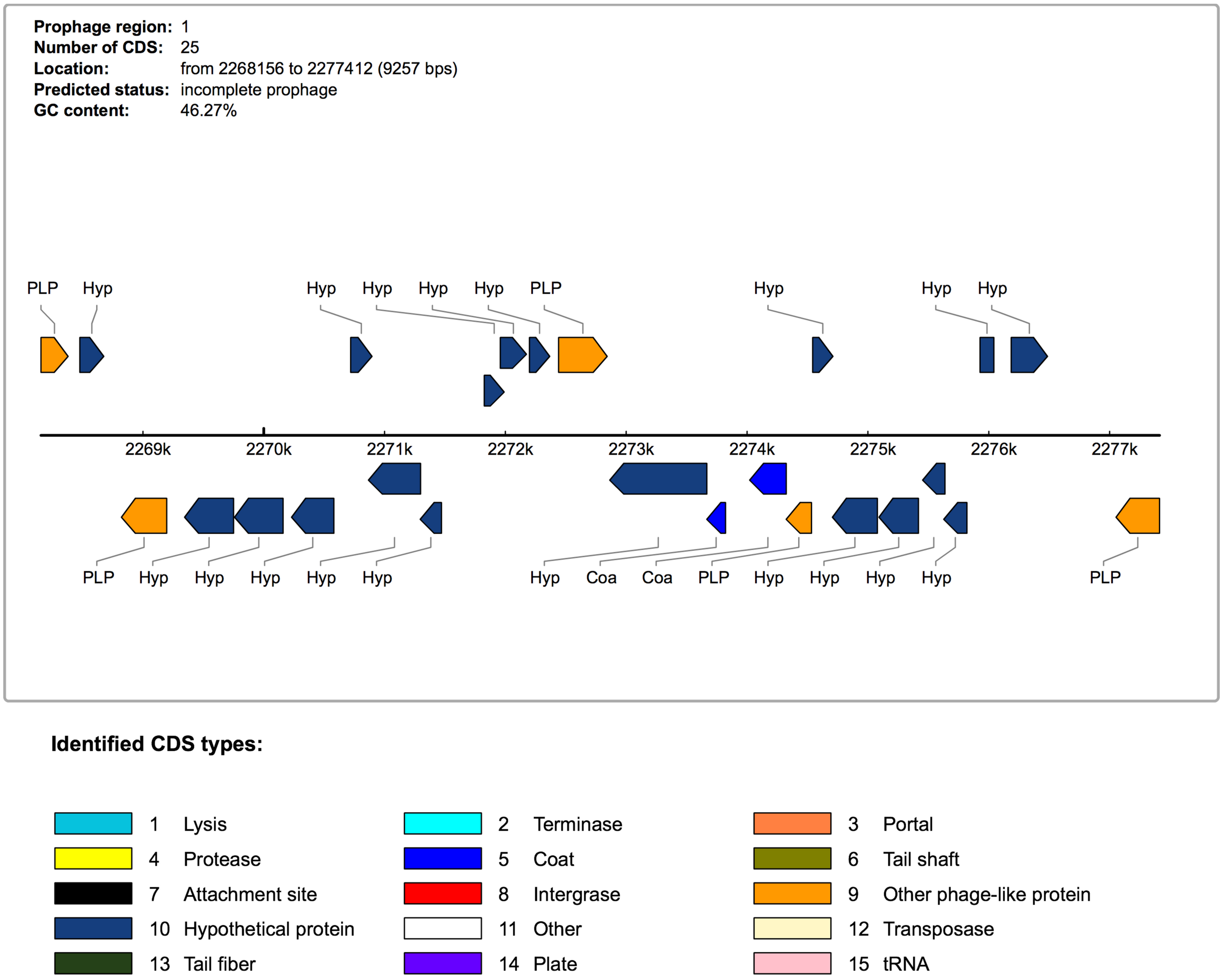

Supplementary Figure 17. Scheme of PHAGE_Prochl_P_SSM3_NC_021559. Possible *Prochlorococcus* phage detected in seawater sample Sw22-2**.**

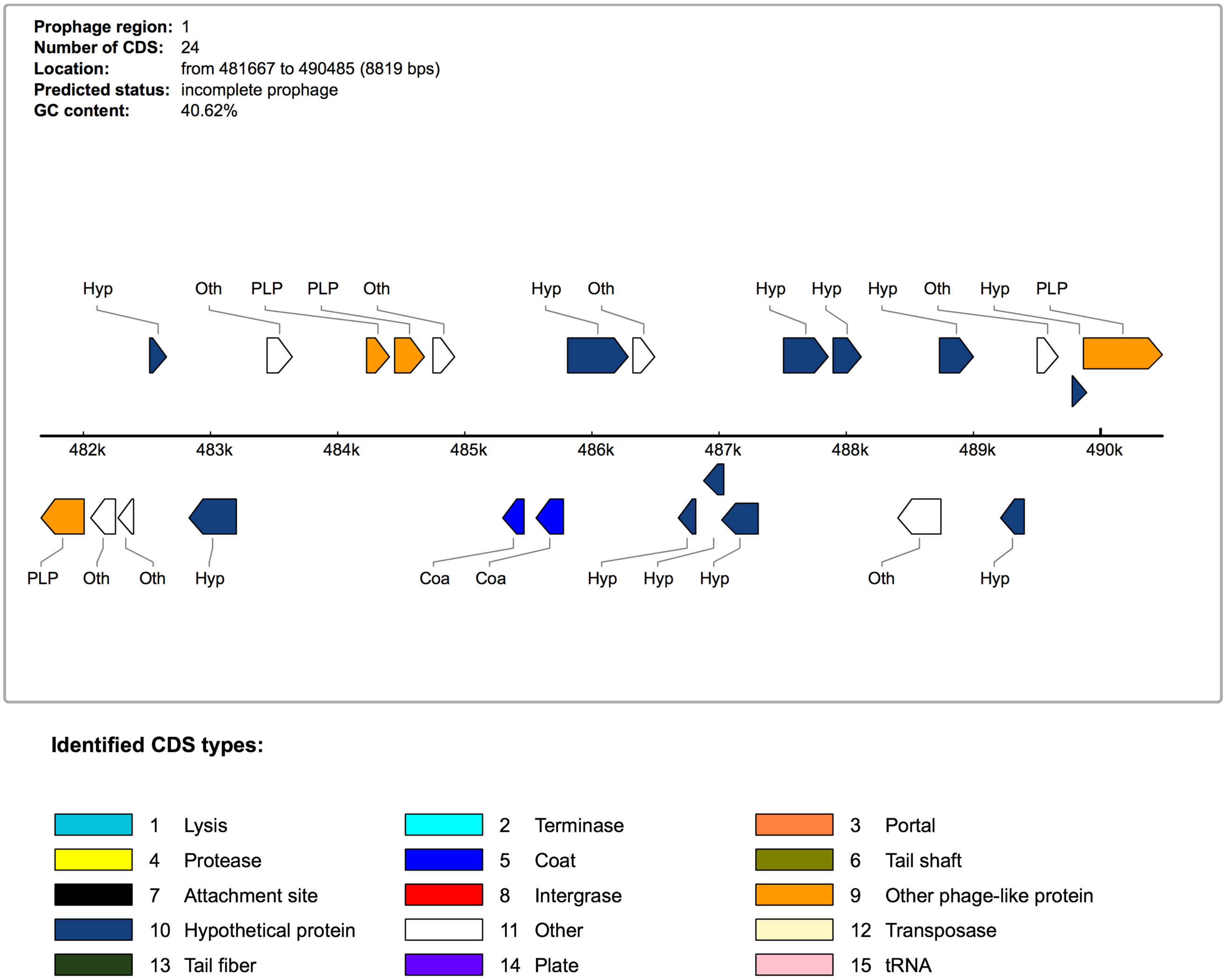

Supplementary Figure 18. Scheme of PHAGE_Synech_S_SM2_NC_015279. Possible *Synechococcus* phage detected in seawater sample Sw22-3.

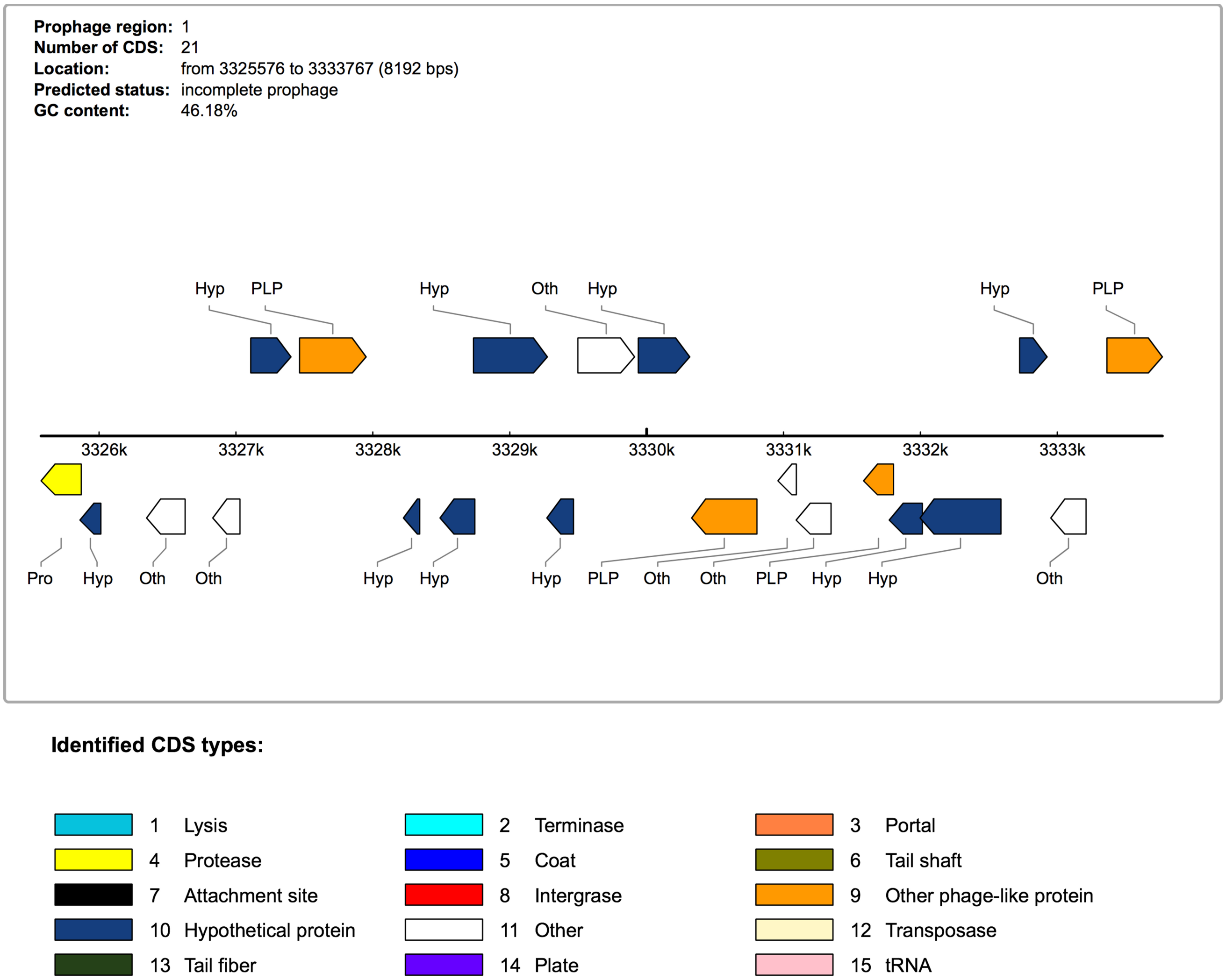

Supplementary Figure 19. Relative abundance of Proteobacteria classes and main bacterial phyla in *M. decatis* and seawater metagenomes. **A** Seawater samples Sw14 and 15, and Sw18 and 22 were pooled and indicated as Sw(14-15) and Sw(18-22), respectively. Healthy *M. decactis* samples Mad14 and 15, and Mad18 and 22 were pooled and indicated as Healthy Coral (14-15) and (18-22), respectively. Bleached *M. decactis* samples MadBle18 and 22 were pooled and indicated as Diseased Coral (18-22). **B** Distribution of the six most abundat bacterial phyla in *M. decatis*. Regression lines for the relative abundances variation are shown at top by *yellow* (Proteobacteria), *orange* (Firmicutes), *pink* (Actinobacteria), *purple* (Bacteroidetes), *green* (Cyanobacteria) and *blue* (Chloroflexi) curves, respectively.

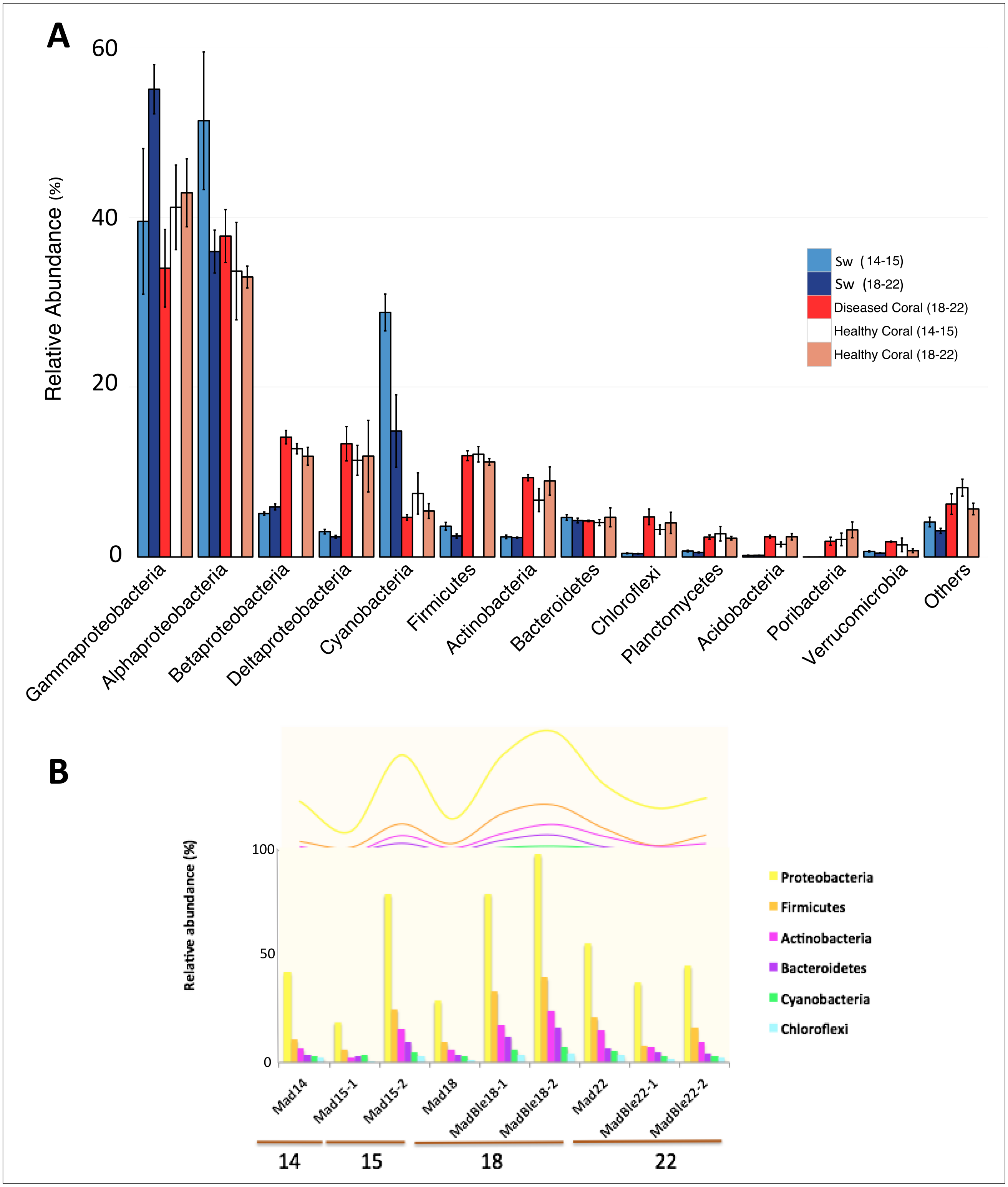
A
Relative Abundance (%)
60
40
20
0
Sw (14-15)
Sw (18-22)
Diseased Coral (18-22)
Healthy Coral (14-15)
Healthy Coral (18-22)
Gammaproteobacteria
Alphaproteobacteria
Betaproteobacteria
Deltaproteobacteria
Cyanobacteria
Firmicutes
Actinobacteria
Bacteroidetes
Chloroflexi
Planctomycetes
Acidobacteria
Poribacteria
Verrucomicrobia
Others
B
Relative abundance (%)
100
50
0
Proteobacteria
Firmicutes
Actinobacteria
Bacteroidetes
Cyanobacteria
Chloroflexi
Mad14
Mad15-1
Mad15-2
Mad18
MadBle18-1
MadBle18-2
Mad22
MadBle22-1
MadBle22-2
14
15
18
22

Supplementary Figure 20. Relative abundance of subsystems in *M. decactis* and seawater metagenomes. Distribution of level 1 SEED subsystems in *M. decatis* and seawater metagenomes. Seawater samples Sw14 and 15, and Sw18 and 22 were pooled and indicated as Sw(14-15) and Sw(18-22), respectively. Healthy *M. decactis* samples Mad14 and 15, and Mad18 and 22 were pooled and indicated as Healthy Coral (14-15) and (18-22), respectively. Bleached *M. decactis* samples MadBle18 and 22 were pooled and indicated as Diseased Coral (18-22).

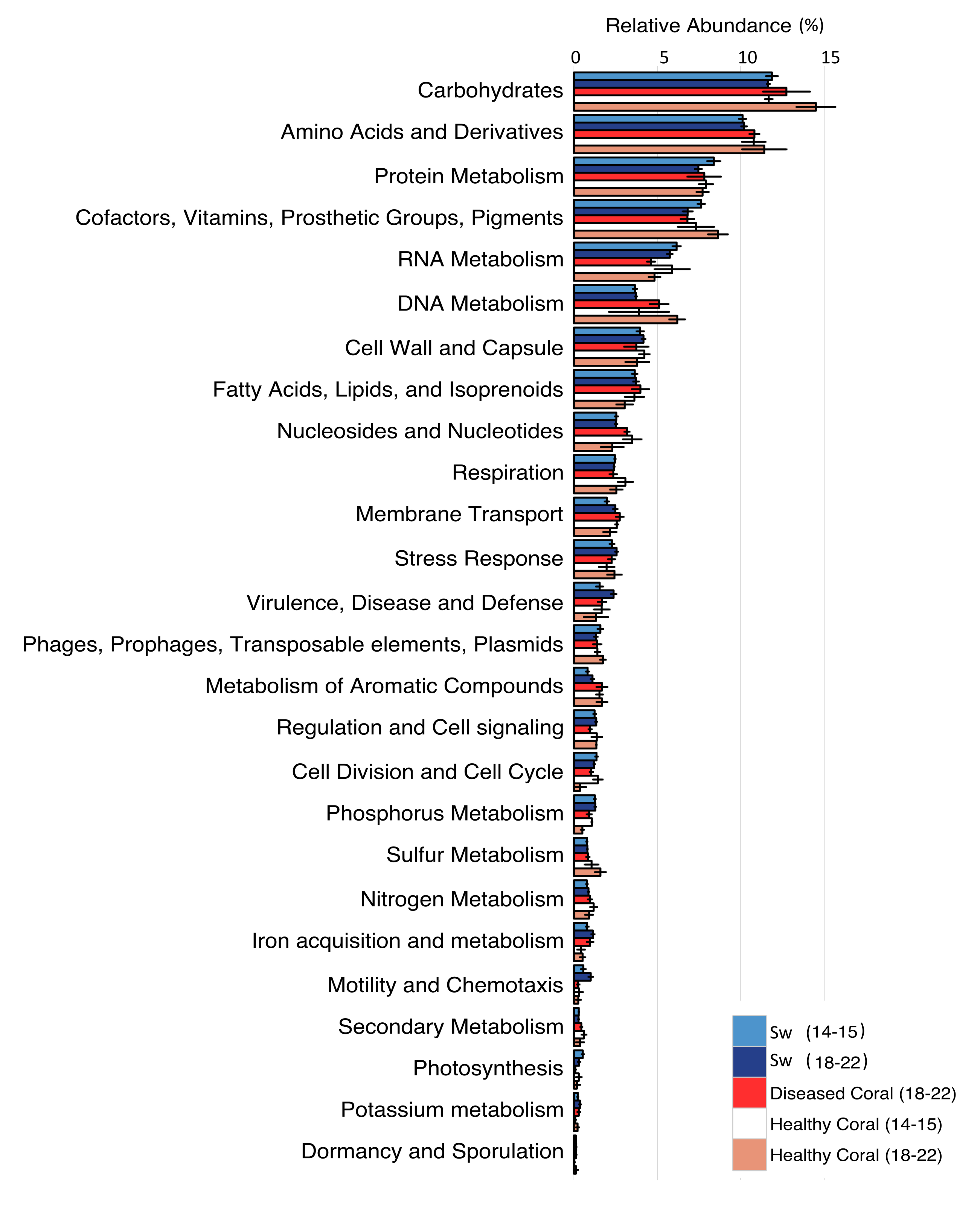

Relative Abundance (%)
0
5
10
15
Carbohydrates
Amino Acids and Derivatives
Protein Metabolism
Cofactors, Vitamins, Prosthetic Groups, Pigments
RNA Metabolism
DNA Metabolism
Cell Wall and Capsule
Fatty Acids, Lipids, and Isoprenoids
Nucleosides and Nucleotides
Respiration
Membrane Transport
Stress Response
Virulence, Disease and Defense
Phages, Prophages, Transposable elements, Plasmids
Metabolism of Aromatic Compounds
Regulation and Cell signaling
Cell Division and Cell Cycle
Phosphorus Metabolism
Sulfur Metabolism
Nitrogen Metabolism
Iron acquisition and metabolism
Motility and Chemotaxis
Secondary Metabolism
Photosynthesis
Potassium metabolism
Dormancy and Sporulation
Sw (14-15)
Sw (18-22)
Diseased Coral (18-22)
Healthy Coral (14-15)
Healthy Coral (18-22)
